# Missed opportunities for HIV testing among those who accessed sexually transmitted infection (STI) services, tested for STIs and diagnosed with STIs: a systematic review and meta‐analysis

**DOI:** 10.1002/jia2.26049

**Published:** 2023-04-26

**Authors:** Kanwal Saleem, Ee Lynn Ting, Andre J. W. Loh, Rachel Baggaley, Maeve B. Mello, Muhammad S. Jamil, Magdalena Barr‐Dichiara, Cheryl Johnson, Sami L. Gottlieb, Christopher K. Fairley, Eric P. F. Chow, Jason J. Ong

**Affiliations:** ^1^ Melbourne Sexual Health Centre Alfred Health Melbourne Victoria Australia; ^2^ Central Clinical School Monash University Melbourne Victoria Australia; ^3^ Global HIV, Hepatitis and STI Programmes World Health Organization Geneva Switzerland; ^4^ Centre for Epidemiology and Biostatistics, Melbourne School of Population and Global Health The University of Melbourne Melbourne Victoria Australia; ^5^ Faculty of Infectious and Tropical Diseases London School of Hygiene and Tropical Medicine London UK

**Keywords:** HIV, HIV testing, missed opportunities, sexually transmitted diseases/diagnosis, sexually transmitted infection, STI testing

## Abstract

**Introduction:**

Of 37.7 million people living with HIV in 2020, 6.1 million still do not know their HIV status. We synthesize evidence on concurrent HIV testing among people who tested for other sexually transmitted infections (STIs).

**Methods:**

We conducted a systematic review using five databases, HIV conferences and clinical trial registries. We included publications between 2010 and May 2021 that reported primary data on concurrent HIV/STI testing. We conducted a random‐effects meta‐analysis and meta‐regression of the pooled proportion for concurrent HIV/STI testing.

**Results:**

We identified 96 eligible studies. Among those, 49 studies had relevant data for a meta‐analysis. The remaining studies provided data on the acceptability, feasibility, barriers, facilitators, economic evaluation and social harms of concurrent HIV/STI testing. The pooled proportion of people tested for HIV among those attending an STI service (*n* = 18 studies) was 71.0% (95% confidence intervals: 61.0–80.1, *I* ^2^ = 99.9%), people tested for HIV among those who were tested for STIs (*n* = 15) was 61.3% (53.9–68.4, *I* ^2^ = 99.9%), people tested for HIV among those who were diagnosed with an STI (*n* = 13) was 35.3% (27.1–43.9, *I* ^2^ = 99.9%) and people tested for HIV among those presenting with STI symptoms (*n* = 3) was 27.1% (20.5–34.3, *I* ^2^ = 92.0%). The meta‐regression analysis found that heterogeneity was driven mainly by identity as a sexual and gender minority, the latest year of study, country‐income level and region of the world.

**Discussion:**

This review found poor concurrent HIV/STI testing among those already diagnosed with an STI (35.3%) or who had symptoms with STIs (27.1%). Additionally, concurrent HIV/STI testing among those tested for STIs varied significantly according to the testing location, country income level and region of the world. A few potential reasons for these observations include differences in national STI‐related policies, lack of standard operation procedures, clinician‐level factors, poor awareness and adherence to HIV indicator condition‐guided HIV testing and stigma associated with HIV compared to other curable STIs.

**Conclusions:**

Not testing for HIV among people using STI services presents a significant missed opportunity, particularly among those diagnosed with an STI. Stronger integration of HIV and STI services is urgently needed to improve prevention, early diagnosis and linkage to care services.

## 1 INTRODUCTION

According to the Joint United Nations Programme on HIV/AIDS (UNAIDS), approximately 37.7 million people were living with HIV (PLHIV) in 2020, including 36 million adults and 1.7 million children; of these, 6.1 million people globally were not aware of their HIV status [1]. Access to early testing is essential for HIV prevention, treatment and linkage to care. Earlier diagnosis and subsequent antiretroviral therapy (ART) initiation significantly decrease HIV‐related morbidity and mortality and the risk of onward transmission, resulting in the improved long‐term health of PLHIV and their communities [2]. Therefore, more efficient and effective ways to reach the UN global targets to diagnose 95% of PLHIV and link them to care are required. To achieve this, countries need to develop a strategic mix of testing approaches; this can include targeted testing based on risk and symptoms [3] and routine testing for people attending clinical services for sexually transmitted infections (STIs).

Individuals with STIs are at an increased risk of transmitting and acquiring HIV due to biological factors and similar high‐risk sexual practices, such as condomless sex or multiple sexual partners [4]. Studies show that detecting and treating STIs may reduce HIV acquisition and transmission [5]. These findings underscore the need for improved routine STI services that include the offer of HIV testing among people tested for STIs (i.e. concurrent HIV/STI testing) at the same visit. Regular concurrent HIV/STI testing for those at higher risk facilitates early HIV diagnosis and might also reduce the onward transmission of HIV or other STIs [6].

In 2007, the World Health Organization (WHO) recommended the routine offer of concurrent HIV testing in all STI services [7] and reinforced this in all subsequent testing guidelines. In 2019, there were further recommendations for testing and retesting for people presenting with a diagnosis or receiving treatment for STIs [8]. This includes dual HIV/syphilis rapid diagnostic tests that can be considered the first test in HIV testing strategies and algorithms in antenatal care settings and for key populations [9]. HIV testing is also recommended to be integrated with other clinical services, including STIs and tuberculosis, to create opportunities for the early diagnosis of co‐infections and increase the uptake of HIV testing among populations at higher risk for HIV infection [10].

Despite these long‐standing global guidelines, HIV testing among people tested for STIs or presenting with STI symptoms in diverse healthcare settings (community‐based services, hospitals, STI clinics and physician/primary care outpatient clinics) remains suboptimal. In 2016, a retrospective US‐based study with participants from 29 states showed that only 43% of the participants diagnosed with an STI in a physician outpatient clinic or emergency department were screened for HIV [11]. Similarly, a Spain‐based study conducted in 2016 reported that HIV testing was conducted among 61% of people diagnosed with other STIs in various settings, including primary care, hospital or clinic, sexual health clinic and medical specialist [12]. Furthermore, data from the paediatric department of Cincinnati Children's Hospital Medical Centre in the United States observed test uptake as low as 3.6% among adolescents diagnosed with an STI [13].

A prior systematic review identified HIV testing interventions among healthcare settings in Europe [14] and another on how incentives could improve HIV/STI testing rates [15]. However, to our knowledge, there are no systematic reviews on concurrent HIV/STI testing uptake across different healthcare settings globally. This systematic review and meta‐analysis aim to synthesize the existing evidence on the routine offer and uptake of HIV testing among people attending an STI service, tested for other STIs, diagnosed with STIs or with STI symptoms. Secondary aims included identifying barriers and facilitators for concurrent HIV/STI testing.

## 2 METHODS

We conducted a systematic review (Prospero: CRD42021231321) that followed the guidelines in the Cochrane handbook for systematic reviews [16] and the PRISMA (Preferred reporting items for systematic reviews and meta‐analyses) guidelines for reporting [17]. Five databases (Ovid MEDLINE, Ovid Embase, Ovid Global Health, EBSCO CINAHL Plus and Web of Science Core Collection), conferences related to HIV (Conference on Retroviruses and Opportunistic Infections, HIV Research for Prevention Conference, International AIDS Conference, International Conference on AIDS and STIs in Africa, US CDC Prevention and British Association for Sexual Health and HIV) and clinical trial registries (clinicaltrials.gov and WHO international clinical trials registry platform) were searched for publications between 1st January 2010 and 5th May 2021 that documented primary data on concurrent HIV/STI testing uptake.

### 2.1 Study eligibility criteria

We included any studies in English and contained data for the number of people tested for HIV among the number of people who attended an STI service, tested for STIs, diagnosed with STIs (chlamydia, gonorrhoea or syphilis) or with symptoms of STIs. As part of our secondary outcomes, we also included studies reporting the acceptability, feasibility, barriers, facilitators, economic evaluation and social harms of concurrent HIV‐STI testing. We excluded duplicated results from the same study or laboratory studies testing HIV diagnostic performance.

### 2.2 Search method and data extraction

Key concepts included in the search strategy were: (1) HIV and STIs; (2) tests and screening; and (3) early diagnosis, missed opportunities. Additional details on the search strategy are included in the online Appendix (Additional file 3: Search Strategy). All studies’ titles and abstracts were independently screened by at least two reviewers (KS, ET and AL) using the inclusion criteria. The full texts of potentially relevant papers were independently screened by at least two reviewers (KS, ET and AL) and any discrepancies were resolved by another researcher (JO). Relevant data related to primary and secondary outcomes were extracted from deduplicated publications. We conducted a qualitative synthesis of factors associated with concurrent HIV/STI testing and classified each attribute using the socio‐ecological model [18]: “individual factors,” “service factors” and “societal factors.”

### 2.3 Statistical analysis

Random‐effects meta‐analysis was used to calculate across‐study pooled proportions of people tested for HIV among those attending an STI service, tested for other STIs or diagnosed with STIs. Pooled proportions and 95% confidence intervals were generated using a Freeman–Tukey–type double arcsine transformation to adjust for variance instability. Statistical heterogeneity between studies was assessed with the *I* ^2^ statistic. Predefined subgroup meta‐analyses were based on the following covariates: country‐income level, type of HIV testing (rapid testing, venepuncture), recruitment site, study population, the latest year of study and region of the world. Funnel plots were generated to assess the possibility of small‐study effects associated with publication bias. Egger's test was performed to confirm the presence of this bias. When publication bias was significant (*p*<0.05), we used a nonparametric trim‐and‐fill analysis to explore the sensitivity of the meta‐analysis results to potentially omitted studies. Random‐effects meta‐regression models using the covariates described above were conducted to examine the association of these variables with the effect size. Adjusted *R* ^2^ is reported for the percentage of variance explained by the subgroups above. All analyses were conducted using Stata, version 17.0 (StataCorp LLC). We evaluated the methodological quality using the Cochrane risk of bias tool for randomized controlled trials, the Newcastle‐Ottawa quality assessment scale for cross‐sectional, cohort and case–control studies, the consolidated health economic evaluation reporting standards checklist for economic evaluation studies and Joanna Briggs Institute critical appraisal checklist for qualitative studies (Additional file 4: Quality Checklists).

## 3 RESULTS

**Figure 1:**
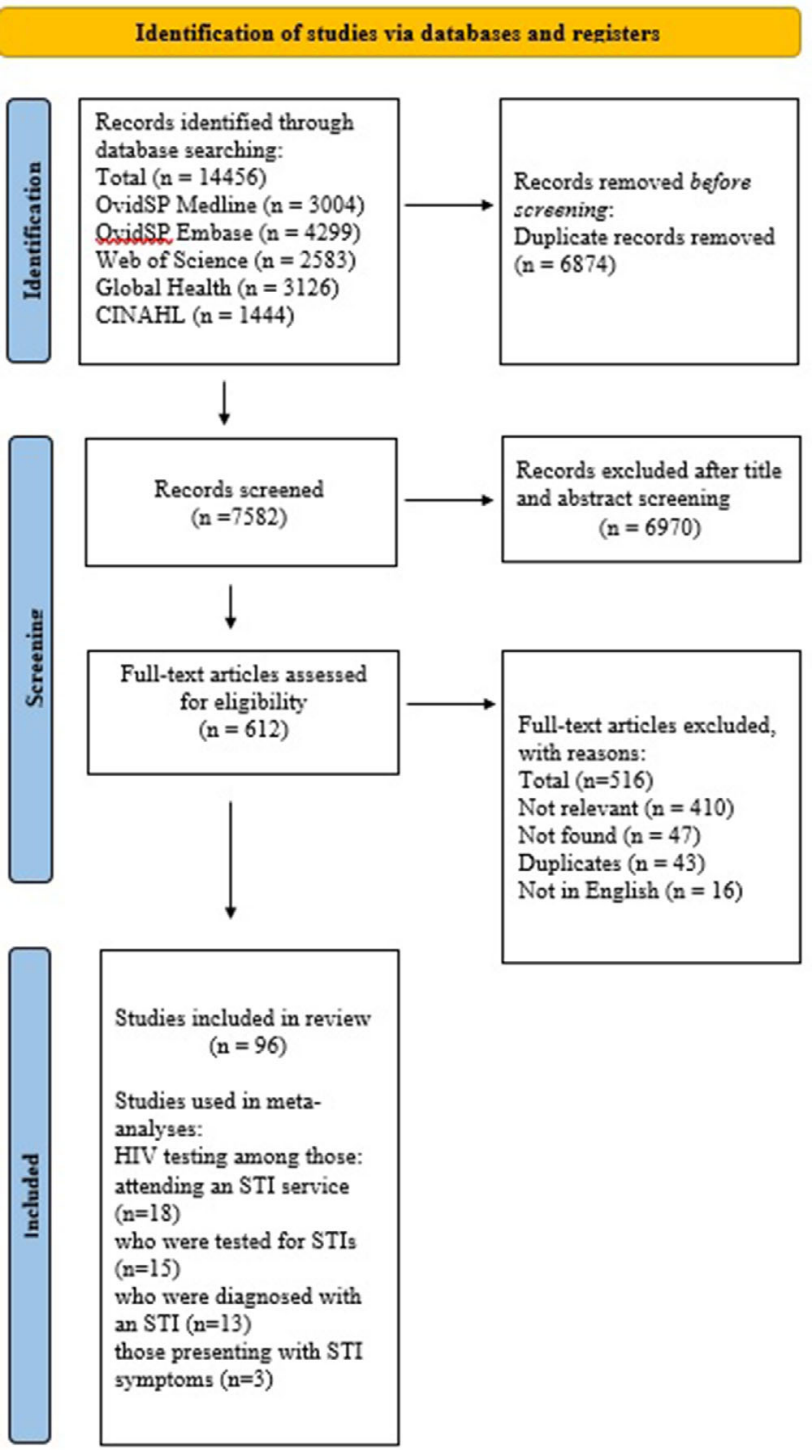
**PRISMA flowchart**.

Of 7582 articles, 612 full texts were examined, and 96 studies were included in our final analysis (Figure 1). Among the 96 studies, 18 studies had relevant data for a meta‐analysis of the proportion of people tested for HIV among those attending an STI service, 15 studies among those tested for STIs, 13 studies among those diagnosed with STI and three studies for people with STI symptoms. The remaining studies provided data related to the secondary outcomes. Table 1 provides an overview of the included studies. In brief, the majority (73%) of studies were from high‐income countries (HICs) (Figure S5), with about a third from general populations (36%) and sexual and gender minorities (i.e. those who do not identify as cisgender heterosexual (30%). (Table S1 provides further details of each included study).

**Table 1:** Summary of included studies (*N* = 96)

	*n* (%)
*Latest year of study* ^a^	
Before 2010	19 (19.8)
2010–2014	40 (41.7)
2015–2021	25 (26.0)
*Type of studies*	
Randomized controlled trial	3 (3.1)
Observational	78 (82.1)
Economic evaluation	3 (3.1)
Qualitative	16 (16.8)
Other	1 (1)
*Region of world*	
Americas	43 (44.7)
African	10 (10.4)
Eastern Mediterranean	1 (1)
Europe	20 (20.8)
South‐East Asia	2 (2)
Western Pacific	
*Country income level* ^b^	20 (20.8)
Low	3 (3)
Lower‐middle	7 (7.1)
Upper‐middle	14 (14.2)
High	72 (73.4)
Not specified	2 (2)
*Study population*	
General population	35 (35.7)
Sexual and gender minorities^c^	29 (29.5)
Youth	11 (11.2)
Sex workers	4 (4)
Other (health providers, inmates, asylum seekers, older people and low‐income women)	18 (18.3)
Not specified	1 (1)
*Testing setting*	
Hospital	8 (6.8)
Outpatient	14 (12)
Emergency department	11 (9.4)
Community‐based facilities (GPs)	9 (7.7)
STI clinic	24 (20.6)
Other (university, home, social settings and migrant health clinic)	14 (12)
Not specified	36 (31)
*HIV test conducted by*	
Self‐testing	9 (7.4)
Nurse	22 (18.1)
Doctor	23 (19)
Counsellor	1 (0.8)
Peer	1 (0.8)
Other	1 (0.8)
Not specified	64 (52.8)

^a^12 studies did not have a clear study year.

^b^Country income level was determined using the World Bank classification [19].

^c^Those who do not identify as cisgender heterosexual.

### 3.1 Percentage of people tested for HIV among those attending a clinic with STI testing service

**Figure 2:**
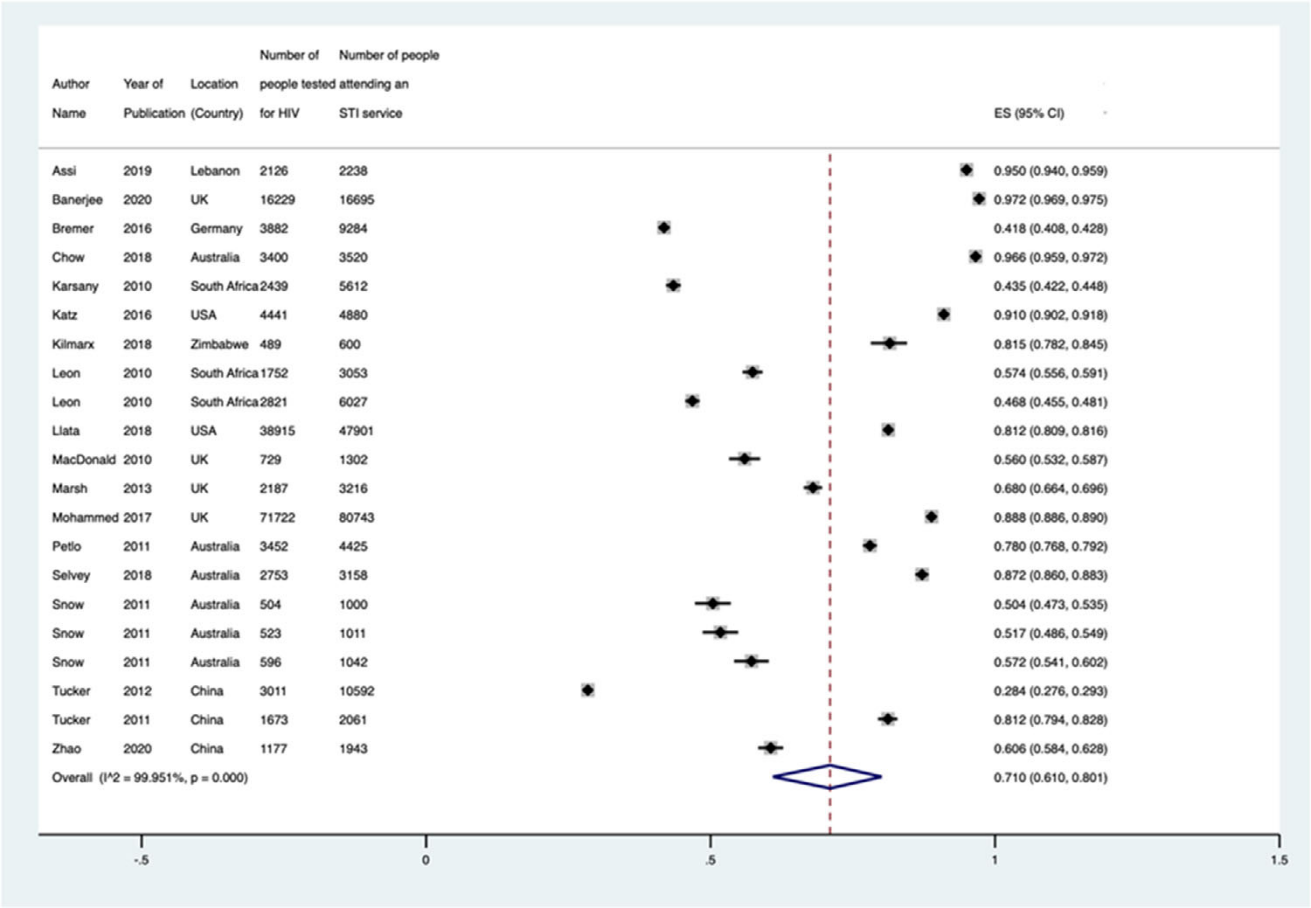
**Forest plot for HIV testing among people attending a clinic with STI testing services**.

Eighteen studies provided 21 estimates for the meta‐analysis (Figure 2 and Table 2) [20, 21, 22, 23, 24, 25, 26, 27, 28, 29, 30, 31, 32, 33, 34, 35, 36, 37]. The pooled percentage of people tested for HIV when attending an STI service was 71.0% (95% CI: 61.0–80.1%). There was no evidence of publication bias (*p* = 0.837) (Figure S1). The meta‐regression analysis revealed that sexual and gender minority populations compared with those not belonging to a sexual and gender minority (AOR 1.37, 95% CI: 1.10–1.71) and recent latest year of study (2010–2014): AOR 1.68 (95% CI: 1.17–2.40); 2015 onwards: AOR 1.46 (95% CI: 1.26–1.29) compared with before 2010, explained most of the heterogeneity (adjusted *R* ^2^ = 91.3%).

**Table 2:** Pooled percentage of people tested for HIV who attended an STI service

	Number of studies	Pooled % of people tested for HIV	*I* ^2^ (*p* value)
Total	18	71.0 (61.0–80.1)	99.95% (<0.001)
Country income level			
High	11	75.4 (66.3–83.4)	99.93% (<0.001)
Middle	7	63.5 (46.4–79.0)	99.89% (<0.001)
Type of HIV testing			
Rapid HIV testing	4	74.5 (52.6–91.2)	99.85% (<0.001)
Venepuncture	4	62.1 (61.0–80.1)	99.88% (<0.001)
Unclear	10	74.3 (60.5–86.0)	99.97% (<0.001)
Study population			
Sexual and gender minorities^a^	7	79.1 (70.8–86.3)	99.73% (<0.001)
Not sexual and gender minorities	11	64.5 (46.5–80.5)	99.97% (<0.001)
Latest year of study			
Before 2010	6	52.3 (40.3–64.1)	99.79% (<0.001)
2010–2014	6	76.9 (64.4–87.3)	99.95% (<0.001)
2015 onwards	6	88.6 (78.0–96.0)	99.77% (<0.001)
Region of the world			
Americas	2	82.3 (81.9–82.6)	–
African	4	57.7 (47.3–67.8)	99.35% (<0.001)
Eastern Mediterranean	1	95.0 (94.0–95.9)	–
Europe	5	73.5 (51.5–90.7)	99.97% (<0.001)
South‐East Asia	0	–	–
Western Pacific	7	67.7 (46.1–85.9)	99.92% (<0.001)

^a^Those who do not identify as cisgender heterosexual.

### 3.2 Percentage of people tested for HIV among those tested for an STI

**Figure 3:**
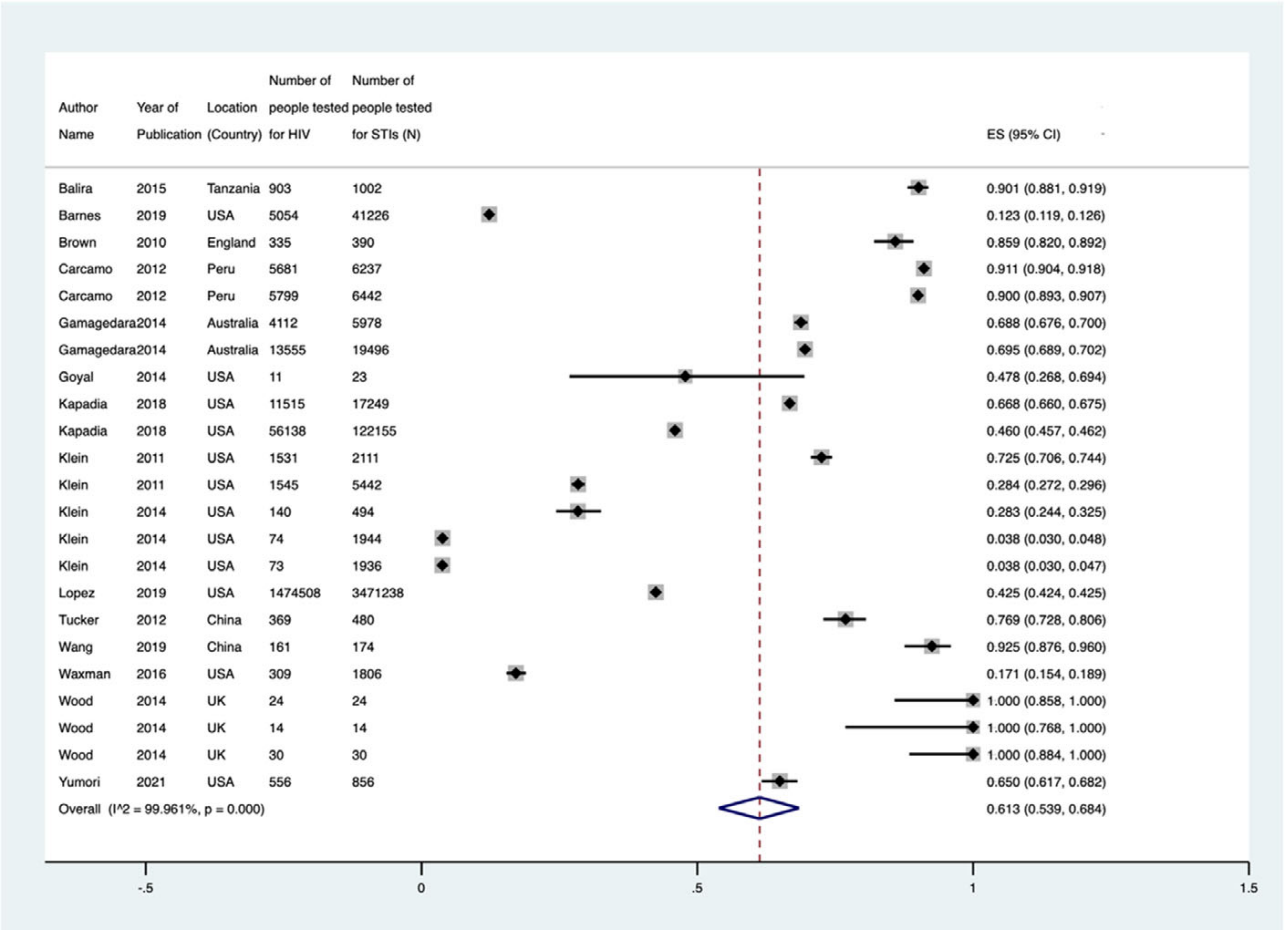
**Forest plot for HIV testing among people tested for STIs**.

Fifteen studies provided 23 estimates for the meta‐analysis (Figure 3 and Table 3) [38, 39, 40, 41, 42, 43, 44, 45, 46, 47, 48, 49, 50, 51, 52]. The pooled percentage of people tested for HIV among those tested for STIs independent of the type of service was 61.3% (95% CI: 53.9–68.4%, *I* ^2^ = 99.96%). There was evidence of publication bias (*p* = 0.032) (Figure S2) with a pooled prevalence of 57.8% (95% CI: 44.8–70.8) when we imputed potentially missing studies. The meta‐regression analysis revealed that studies from middle‐income countries compared with HICs (AOR 2.14, 95% CI: 1.44–3.17) and regions of the world (Europe: AOR 2.04 [95% CI: 1.22–3.42]; Western Pacific: AOR 1.60 [95% CI: 1.04–2.44]) compared with the region of the Americas, explained most of the heterogeneity (adjusted *R* ^2^ = 70.5%).

**Table 3:** Pooled percentage of people tested for HIV who were tested for an STI

	Number of studies	Pooled % of people tested for HIV	*I* ^2^ (*p* value)
Total	15	61.3 (53.9–68.4)	99.96% (<0.001)
Country income level			
High	11	51.1 (43.6–58.4)	99.96% (<0.001)
Middle	4	88.6 (85.8–91.2)	94.44% (<0.001)
Type of HIV testing			
Rapid HIV testing	2	91.6 (89.7–93.4)	–
Venepuncture	8	59.3 (43.4–74.3)	98.98% (<0.001)
Unclear	5	57.0 (44.3–69.2)	99.76% (<0.001)
Recruitment site			
STI clinic	5	75.0 (71.2–78.7)	95.61% (<0.001)
Hospital outpatients	1	65.0 (61.7–68.2)	–
Emergency department	4	18.5 (8.7–30.9)	99.70% (<0.001)
General Practice	1	65.0 (61.7–68.2)	–
Online	1	100 (76.8–100)	–
Study population			
Sexual and gender minorities^a^	2	98.4 (93.0–100)	53.40% (<0.001)
Not sexual and gender minorities	13	52.6 (44.6–60.6)	99.97% (<0.001)
Latest year of study			
Before 2010	5	59.8 (32.6–84.0)	99.94% (<0.001)
2010–2014	4	79.1 (60.0–93.4)	99.82% (<0.001)
2015 onwards	6	48.4 (38.3–58.5)	99.98% (<0.001)
Region of the world			
Americas	9	42.5 (33.6–51.5)	99.97% (<0.001)
African	1	90.1 (88.1–91.9)	–
Eastern Mediterranean	0	–	–
Europe	2	97.9 (86.9–100)	–
South‐East Asia	0	–	–
Western Pacific	3	75.7 (71.6–79.6)	96.18% (<0.001)

^a^Those who do not identify as cisgender heterosexual.

### 3.3 Percentage of people tested for HIV among those diagnosed with STIs

**Figure 4:**
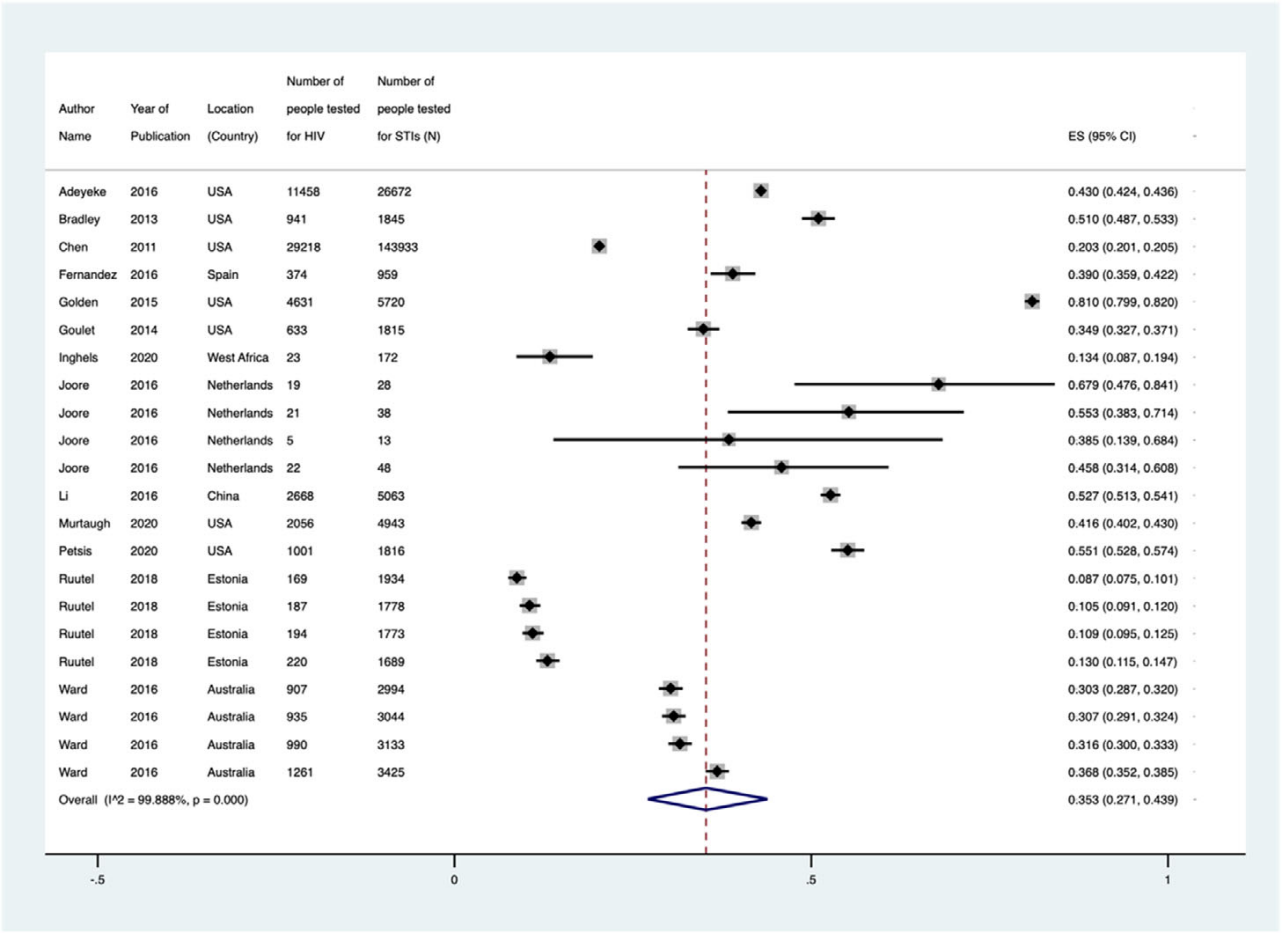
**Forest plot for HIV testing among those diagnosed with an STI**.

Thirteen studies provided 22 estimates for the meta‐analysis (Figure 4 and Table 4) [2, 11, 12, 53, 54, 55, 56, 57, 58, 59, 60, 61, 62]. The pooled percentage of people tested for HIV among those diagnosed with an STI was 35.3% (95% CI: 27.1–43.9, *I* ^2^ = 99.89%). There was no evidence of publication bias (*p* = 0.088, Figure S3). We could only run the univariable analysis for the meta‐regression analysis as there were insufficient observations for the multivariable analysis. We found that sexual and gender minorities (OR 1.62, 95% CI: 1.16–2.25) compared with non‐sexual and gender minorities were more likely to be tested for HIV among those diagnosed with STIs (adjusted *R* ^2^ = 32.5%).

**Table 4:** Pooled percentage of people tested for HIV among those diagnosed with STIs

	Number of studies^a^	Pooled % of people tested for HIV	*I* ^2^ (*p* value)
Total	13	35.3 (27.1–43.9)	99.89% (<0.001)
Country income level			
High	11	35.6 (27.1–44.6)	99.89% (<0.001)
Mixed	2	51.3 (49.9–52.6)	–
Type of HIV testing			
Rapid HIV testing	1	39.0 (35.9–42.2)	–
Venepuncture	2	36.7 (29.2–44.5)	98.94% (<0.001)
Unclear	10	34.6 (24.3–45.7)	99.92% (<0.001)
Recruitment site			
STI clinic	2	50.5 (49.2–51.8)	–
Hospital outpatients	1	39.0 (35.9–42.2)	–
General Practice	4	40.7 (34.6–47.0)	97.77% (<0.001)
Study population			
Sexual and gender minorities^b^	1	81.0 (79.9–82.0)	–
Not sexual and gender minorities	12	32.7 (26.2–39.7)	99.82% (<0.001)
Latest study year			
Before 2010	2	23.5 (23.3–23.7)	–
2010–2014	9	37.8 (26.9–49.4)	99.80% (<0.001)
2015 onwards	3	25.4 (2.8–60.1)	–
Region of the world			
Americas	7	46.7 (29.9–63.8)	99.96% (<0.001)
African	1	13.4 (8.7–19.4)	–
Eastern Mediterranean	0	–	–
Europe	3	26.7 (18.3–36.0)	98.44% (<0.001)
South‐East Asia	0	–	–
Western Pacific	1	32.3 (29.4–35.4)	–

^a^Some categories may not add up to the total number of studies because of missing data.

^b^Those who do not identify as cisgender heterosexual.

### 3.4 People with STI symptoms

**Figure 5:**
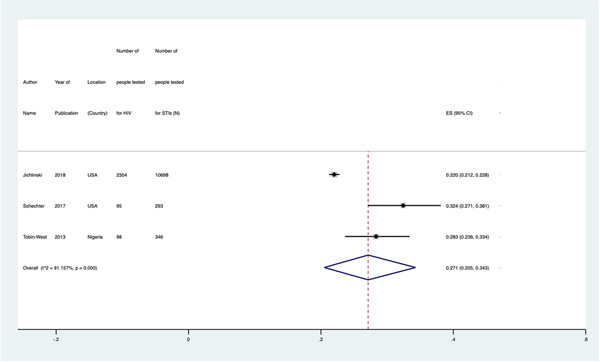
**Forest plot for HIV testing among those with STI symptoms**.

Only three papers provided estimates for HIV testing among people presenting with STI symptoms (Figure 5) [63, 64, 65]. The pooled percentage was 27.1% (20.5–34.3), *I* ^2^ = 91.16 (*p* = 0.017). There was no evidence of publication bias (*p* = 0.269, Figure S4).

### 3.5 Factors associated with concurrent HIV/STI testing

The barriers and facilitators to concurrent HIV/STI testing can be broadly classified into individual and service factors. At an individual level, attitudes or perceptions, fear and knowledge were attributes identified. On a service level, these included the provision of services, ease of access, stigmatizing features, privacy and confidentiality, and bureaucracy. Further details are provided in the online Appendix, but we summarize the key findings below.

#### 3.5.1 Individual‐level barriers

Three studies of varying population types (clients with STIs, clients attending an STI clinic and people attending a genitourinary medicine clinic) elaborated that a low perceived susceptibility to HIV acted as deterrence for testing; this included people who felt that they had no or little exposure to HIV risk factors [29, 37, 48]. Among those tested for STIs, two studies mentioned that most participants chose not to accept an HIV test as they had previously been tested [24, 37]. Fear of HIV testing (including the fear of result disclosure, needle phobia and fear of financial costs) was a common reason for refusing the test among all clients [24, 29, 66]. The stigma associated with HIV testing among clients attending a Nigerian STI service or a clinic was accompanied by the refusal and provision of HIV testing, although this was untrue among clients attending an STI Clinic in Urban China [48, 65, 66]. For clients attending an STI service or being tested for STIs, insufficient knowledge around HIV testing (unaware of testing methods and where testing can be performed) was another commonly cited reason for refusing HIV testing [31, 67]. Overall, independent of whether clients were clients with STIs or attending an STI service/GP Clinic, the individual barriers to testing were similar.

#### 3.5.2 Individual‐level facilitators

Among clients tested for HIV while attending an STI service, integration of HIV counselling and education (e.g. peer‐based education targeting youth, provider‐initiated testing and counselling) into HIV testing was associated with increased HIV testing uptake [37, 68].

#### 3.5.3 Service‐level barriers

In a Dutch study by Moore et al. [69], a reason for not conducting concurrent HIV testing was the concern among health providers that clients would not be able to afford additional HIV testing when presenting for STI testing. Other forms of deterrence from offering HIV testing to clients presenting for STI testing, as reported by health providers, included insufficient time during consultations, low perceived HIV risk by the clinician and having yet to establish a relationship with new clients [66]. In some testing sites in the United States, clients presenting for STI testing were not offered HIV testing simply because of the lack of the offer to test for both HIV and STI in the same visit [69].

#### 3.5.4 Service‐level facilitators

Service factors that improved the ease of access to HIV testing among clients who were tested for STI included the implementation of a dual HIV and syphilis testing strategy [70], express testing services for lower‐risk individuals [42] and convenient testing locations [67]. Internet‐based HIV and STI testing, either through self‐collection or allowing clients to present to designated specimen collection sites, integrated with existing clinic‐based services, can increase HIV testing rates [71]. Clients being tested or diagnosed with STIs at both GP and STI clinics also felt that routine offer of HIV testing would greatly increase testing uptake [27, 67]. Raising awareness of sexual health in a non‐judgemental and professional manner while maintaining confidentiality was reported to increase trust in the healthcare professional and improve the acceptability of HIV testing [67]. Additionally, national policies recommending concurrent HIV testing with STI testing can be effective among female sex workers in Uganda [72].

Where available, we summarized the HIV positivity, sub‐population and recruitment site for each study population in Table S2. HIV positivity varied widely and ranged from 0.2% to 56.49% depending on the country and study population. We examined the pairwise Pearson's correlation between HIV positivity and concurrent testing but did not find any correlation with HIV testing among those attending an STI service (correlation –0.457, *p* = 0.135), or among those tested for STI (corr 0.483, *p* = 0.188). The risk of bias assessments is provided in the online Appendix (pp. 33–52).

## 4 DISCUSSION

This systematic review and meta‐analysis summarized the percentage of HIV testing among those who attended an STI service (71.0%), tested for STIs (61.3%), diagnosed with an STI (35.3%) or had symptoms of an STI (27.1%). To our best knowledge, this is the first attempt to collate these data to highlight the current missed opportunities for HIV testing among those already engaged in care and potentially at higher risk of HIV. Thus, strengthening strategies to improve HIV testing in these settings could help reach the UNAIDS target of diagnosing 95% of people living with HIV. Strengthening the integration of HIV and STI testing through health services is not only important for targeting HIV testing and increasing efficiencies but also for achieving broader goals within the WHO global health sector strategy [73] and the sustainable development goals to eliminate communicable diseases by 2030 [74].

Improving access points to testing could decrease the HIV testing gap. As a minimum, people who are tested for other STIs (especially if they have STI symptoms or an STI diagnosis) should be offered HIV testing, and vice versa, as the risk factors for STIs and HIV often overlap. Our study found that the testing location influenced the level of concurrent HIV/STI testing. While concurrent HIV/STI testing was similarly high in STI clinics (75.0%), hospital outpatient clinics (65%) and general practice (65%), the opposite was found in emergency departments (18.5%). This lower rate of testing in emergency departments is consistent with a systematic review of HIV testing in low‐resource settings, suggesting missed opportunities for better integration of HIV testing into emergency departments [75]. This could include routinely offering HIV testing to all clients being tested for other STIs (opt‐out) [76, 77], improving access to HIV/syphilis dual testing or multiplex HIV/STI testing platforms, ensuring robust systems for follow‐up, and providing education and training to the health workforce in line with the WHO and national testing guidelines.

This review found poor concurrent HIV/STI testing among those already diagnosed with an STI (35.3%) or who had symptoms of STIs (27.1%). We also note the significant variation of concurrent HIV/STI testing among those tested for STIs according to country income level and region of the world. This is despite the WHO recommendation for the routine offer of HIV testing since 2007 (provider‐initiated HIV testing) as a standard component of medical care for clients attending health facilities in high HIV burden settings and for all people with STIs in all settings [39] and for testing and retesting for people presenting with a diagnosis or receiving treatment for STIs [8]. There may be a few potential reasons for these observations. First, differences in national STI‐related policies could impact the uptake of concurrent HIV/STI testing, but this was beyond the scope of the present study. Second, despite the intention to provide concurrent HIV/STI testing, there may be a lack of standard operation procedures (such as reflexive HIV testing with an offer of STI tests) [46]. Third, there may be clinician‐level factors, such as not offering an HIV test unless clients are perceived to be at risk for HIV [78, 79]. Risk‐based screening is highly dependent on clinician time, skill, relationship with clients and client readiness to disclose sexual practices which they perceive they might be judged for. Fourth, there may be poor awareness and adherence to HIV indicator condition‐guided HIV testing (which includes STIs as an indicator) [80]. Finally, there may be an additional stigma associated with HIV, compared to other curable STIs, that may result in a reluctance of providers to offer or for clients to accept HIV testing, even when they were already diagnosed with an STI or suspected to have an STI [81]. However, it is clear that any positive STI test is a marker of risk, and clients (regardless of self‐identification with an “at‐risk” key population group) may often be diagnosed with multiple STIs in the same visit [82, 83, 84]. This further highlights that healthcare worker training, standard operating procedures and resourcing are critical to support concurrent HIV/STI testing.

Among the included studies, there were several situations where concurrent HIV/STI testing was high. First, it was high in studies using the routine offer of HIV testing among STI clients [31, 36, 85, 86]. The routine offer of HIV testing in antenatal settings has been implemented successfully in many countries for more than a decade [87, 88]. In South Africa, the proportion of new STI clients being tested for HIV significantly increased from 42.6% to 56.4% following the universal routine offer of testing [86]. Second, we found settings that had rapid point‐of‐care HIV testing available markedly increased concurrent HIV/STI testing. Rapid testing for HIV/syphilis has high acceptability among clients [89, 90, 91, 92, 93] and could decrease anxiety related to waiting for results, increase convenience and provide greater confidentiality [89, 92]. Third, when HIV testing was integrated into standard STI care protocols, this delivered more consistent performance across clinics [86]. In China, uptake of routine offer of dual HIV/syphilis rapid testing was significantly higher when compared to isolated HIV testing at STI clinics and voluntary counselling and testing clinics [36]. In the United States, the uptake of HIV testing at the time of STI diagnosis/treatment among MSM with bacterial STIs was significantly increased from 62% to 76% following an intervention where all MSM diagnosed with STIs and their partners were offered HIV testing [55]. Fourth, involving nurses in conducting HIV tests and providing HIV chronic disease care and education could increase HIV testing uptake among STI clients [51, 85, 86]. In Australia, HIV testing rates among HIV‐negative MSM significantly increased from 41% to 47% after an STI nurse was introduced into general practice clinics [94]. The authors hypothesized that medical doctors were more willing to initiate HIV testing when nurses were able to share tasks of collecting samples and performing tests, and that nurses could spend more time with clients and thus were more likely to adhere to testing guidelines [94]. This practise is already commonplace outside HICs with many HIV and STI services already being fully nurse‐led and in community settings where trained lay providers often conduct HIV testing.

There was limited information regarding the cost‐effectiveness of concurrent HIV/STI testing beyond dual HIV/syphilis testing among antenatal populations where there is evidence of its cost‐effectiveness [95]. An economic evaluation of universal HIV screening in STI clinics in the United States reported that identifying clients with HIV in STI clinics was more cost‐effective and could even be cost‐saving compared with identifying clients with HIV in hospital inpatients [96]. People with HIV who attended STI clinics were more likely to have higher CD4 counts at the time of diagnosis, allowing for earlier ART initiation [96]. In terms of staff resources, a study in South Africa reported that it was efficient for STI nurses to integrate HIV screening into their consultations [27]. This shift in responsibilities of STI nurses was achieved with relatively short training and by slightly extending their consultation time. A nurse‐led express “Test and Go” HIV/STI testing service for MSM in Melbourne also effectively reduced consultation costs of seeing these men [23]. A modelling analysis of implementing HIV/syphilis dual testing among key populations in Vietnam reported that annual or biannual dual testing could be cost‐effective [97]. Further studies of the cost‐effectiveness of integrating HIV screening with STI testing in a range of settings, especially in low and middle‐income countries, would be helpful to support decision‐making.

The strength of this study is that we systematically reviewed the literature to synthesize knowledge on concurrent HIV/STI testing across a range of settings. This highlighted missed opportunities for HIV testing among individuals at higher risk of infection, specifically those with STI symptoms or an STI diagnosis. Our study had some limitations. First, we only included published data, and most were from an HIC setting, especially from the United States. Therefore, our findings may not be generalizable to LMICs or in settings with a high HIV burden. Second, our search strategy included a third concept related to “early diagnosis, missed opportunities” as using only two concepts (“HIV and STIs” and “Test and Screening”) resulted in too many studies to screen (>100,000). However, this approach may miss relevant studies. Third, we found significant heterogeneity in our meta‐analysis not explained by sampling variability alone. While our meta‐regression analyses identified most of this was due to being a member of a sexual and gender minority group, the latest year of the study, the remaining heterogeneity may be from unmeasured confounders between studies related to patient population characteristics (e.g. background HIV risk, distribution of socio‐economic status), recruitment methods, service‐level factors (e.g. need to pay for STI or HIV testing, type of STIs tested [syphilis only testing compared with services offering syphilis, chlamydia and gonorrhoea testing]) and provider‐level factors (e.g. perception of the need to test clients, time and cost constraints). Nevertheless, our study findings highlight high proportions of missed opportunities to test for HIV. Finally, this review of published literature, although indicating current practices and gaps, may not reflect broad practice, and more work is needed to assess the programme implementation landscape. Published studies may prioritize services that are currently understating some level of HIV testing and/or efforts to increase or improve efficiencies and there may be even greater gaps and missed opportunities, including in LMIC. Data from the Global AIDS Monitoring report that 16% of reporting countries 31/194 in 2021 had a policy of offering dual HIV/syphilis testing for key populations. However, the extent to which this is implemented is not reported. Finally, although commonly used, there may be limitations related to arcsine‐based transformations for meta‐analysis, including a possible violation of the assumption that each study's underlying true transformed proportion follows a normal distribution across studies, or a complicated form of back‐transformation to the original proportion scale [98].

## 5 CONCLUSIONS

In conclusion, we identified significant gaps in concurrent HIV/STI testing globally, specifically among people diagnosed with an STI. We suggest better integration of HIV and STI services, particularly routinely offering HIV testing to all people with STI diagnosis and symptoms, to increase HIV diagnosis in this population at higher HIV risk.

## COMPETING INTERESTS

All authors declare they do not have any competing interests.

## AUTHORS’ CONTRIBUTIONS

JJO designed the research study. JJO, KS, ELT and AJWL performed the research and analysed the data. KS, ELT, AJWL, ML, RB, MBM, MSJ, MBD, CJ, SLG, EPFC, CKF and JJO wrote the paper.

## DISCLAIMER

Some of the authors are present or former staff members of the World Health Organization. The authors alone are responsible for the views expressed in this publication and they do not necessarily represent the views, decisions, or policies of the institutions with which they are affiliated.

## Supporting information


**Figure S1**: Funnel plot for HIV testing among people attending an STI service
**Figure S2**: HIV testing among people tested for STIs
**Figure S3**: Funnel plot of those diagnosed with an STI
**Figure S4**: Funnel plot for people with STI symptoms
**Figure S5**: World map of included studies

Supporting Information

Supporting Informatiom

Supporting Information

## Data Availability

The data that support the findings of this study are available from the corresponding author upon reasonable request.
