# Supplementary material for: Missed opportunities for HIV testing among those who accessed sexually transmitted infection (STI) services, tested for STIs and diagnosed with STIs: a systematic review and meta‐analysis
Source: J Int AIDS Soc. 2023 Apr 26;26(4):e26049. doi: 10.1002/jia2.26049 (PMC10131090; doi:10.1002/jia2.26049)
Supplement: Supplementary file 1 — Figure S1: Funnel plot for HIV testing among people attending an STI service Figure S2: HIV testing among people tested for STIs Figure S3: Funnel plot of those diagnosed with an STI Figure S4: Funnel plot for people with STI symptoms Figure S5: World map of included studies [file JIA2-26-e26049-s002.docx]

**
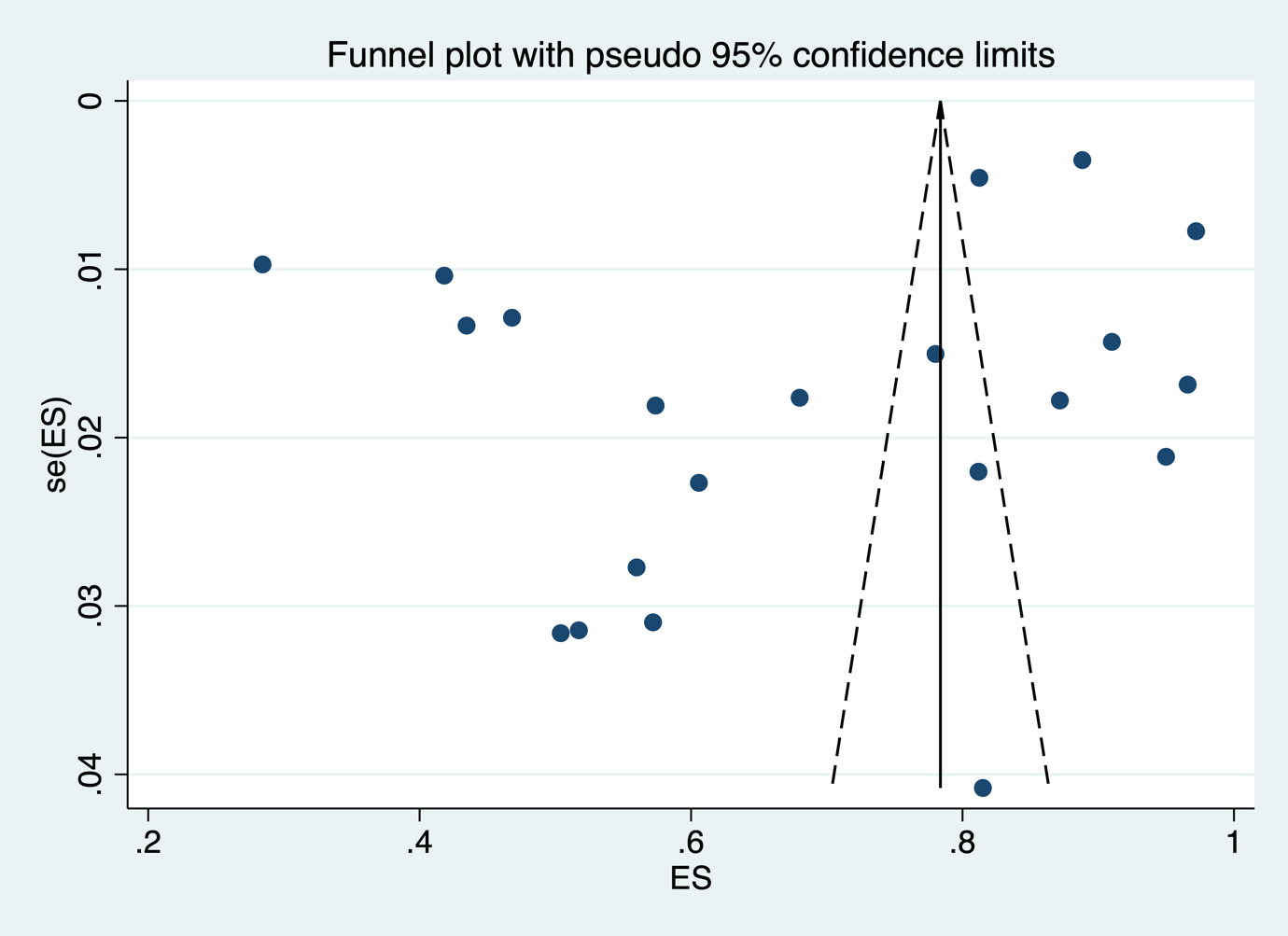
**

**Supplementary Figure 1 Funnel plot for HIV testing among people attending an STI service**

**Egger’s test, p=0.837**

**
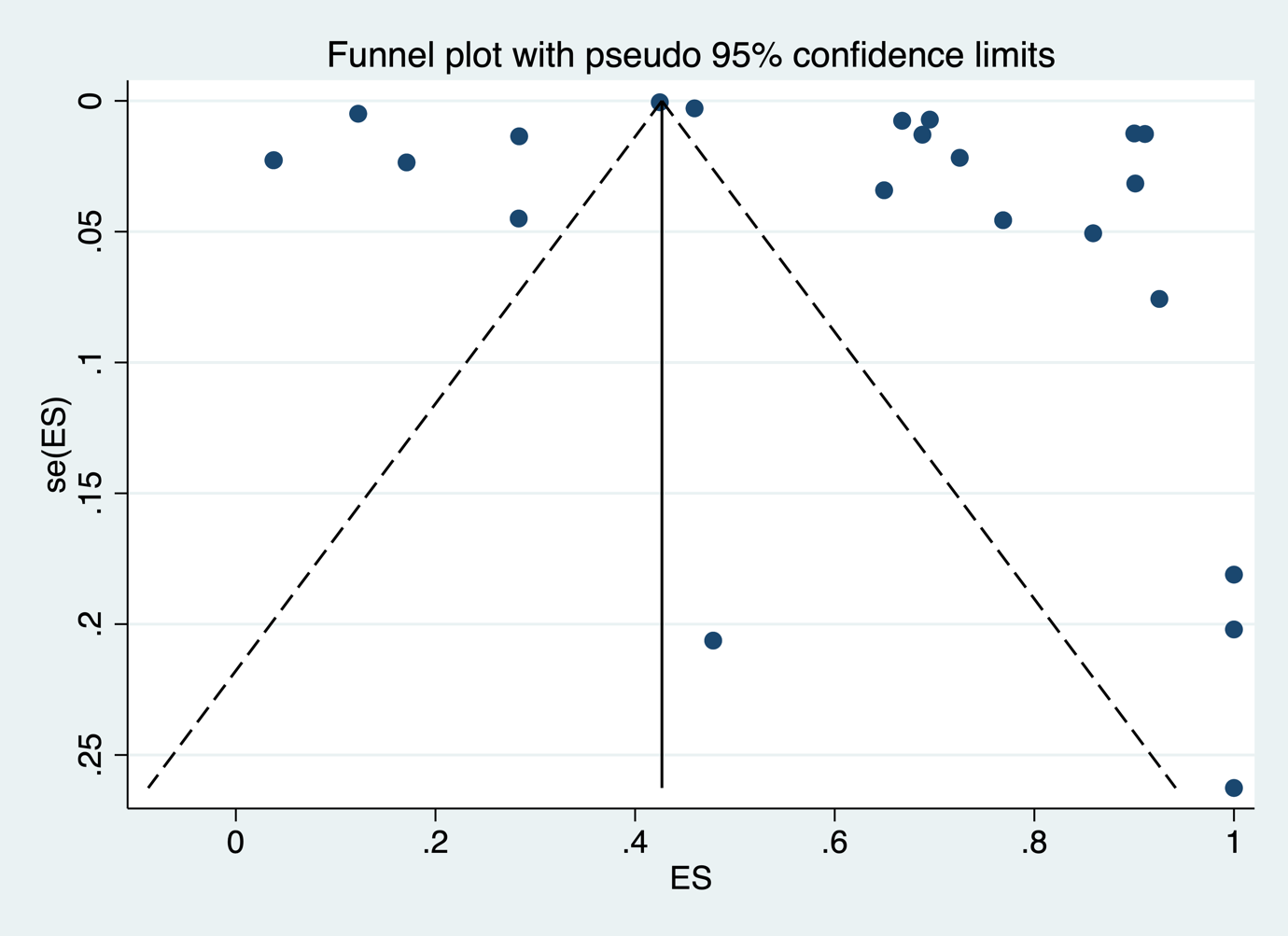
**

**Supplementary Figure 2 HIV testing among people tested for STIs**

**Egger’s test 0.032**

**
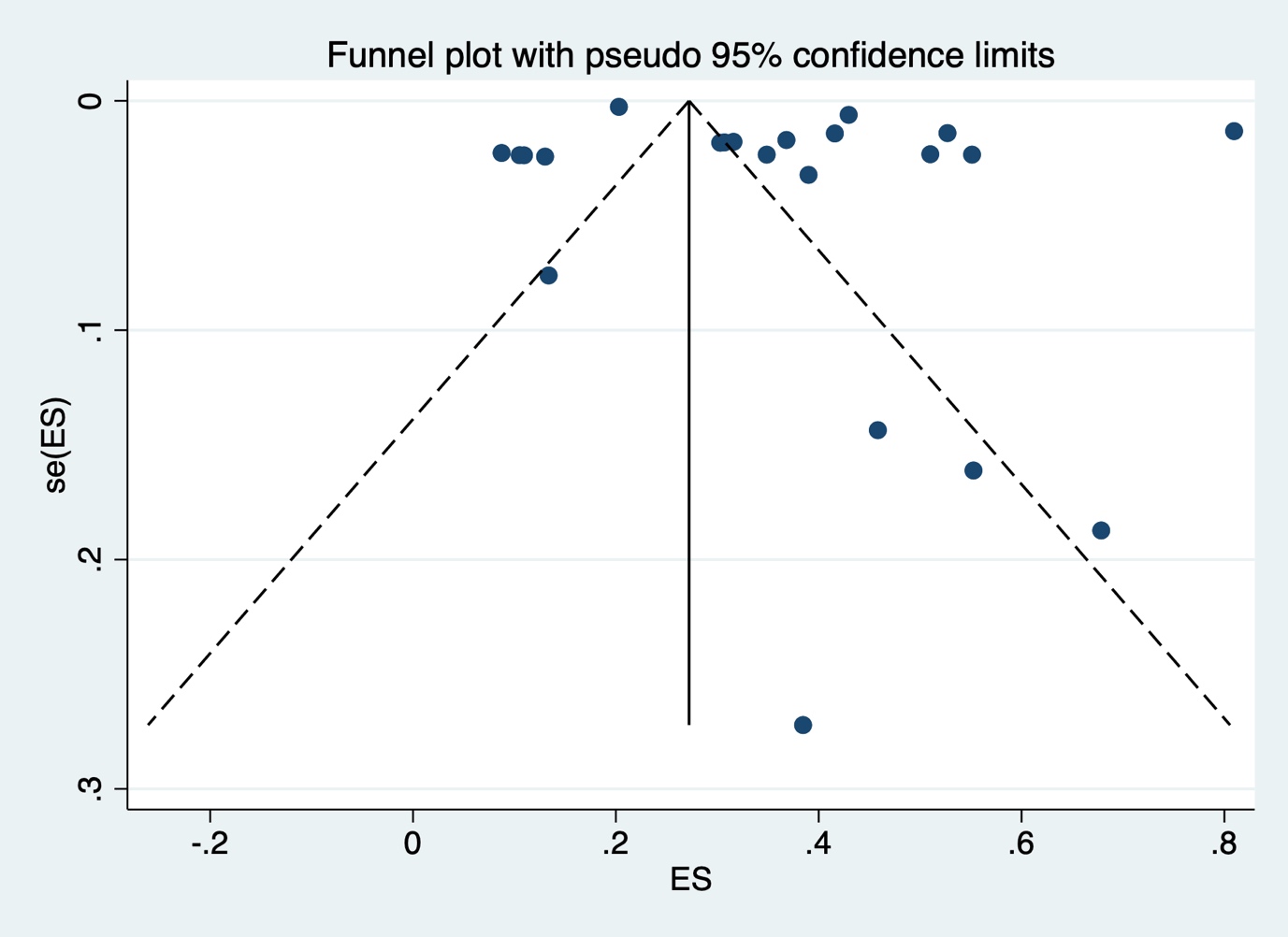
**

**Supplementary Figure 3 Funnel plot of those diagnosed with an STI**

**Egger’s test, p=0.088**

**
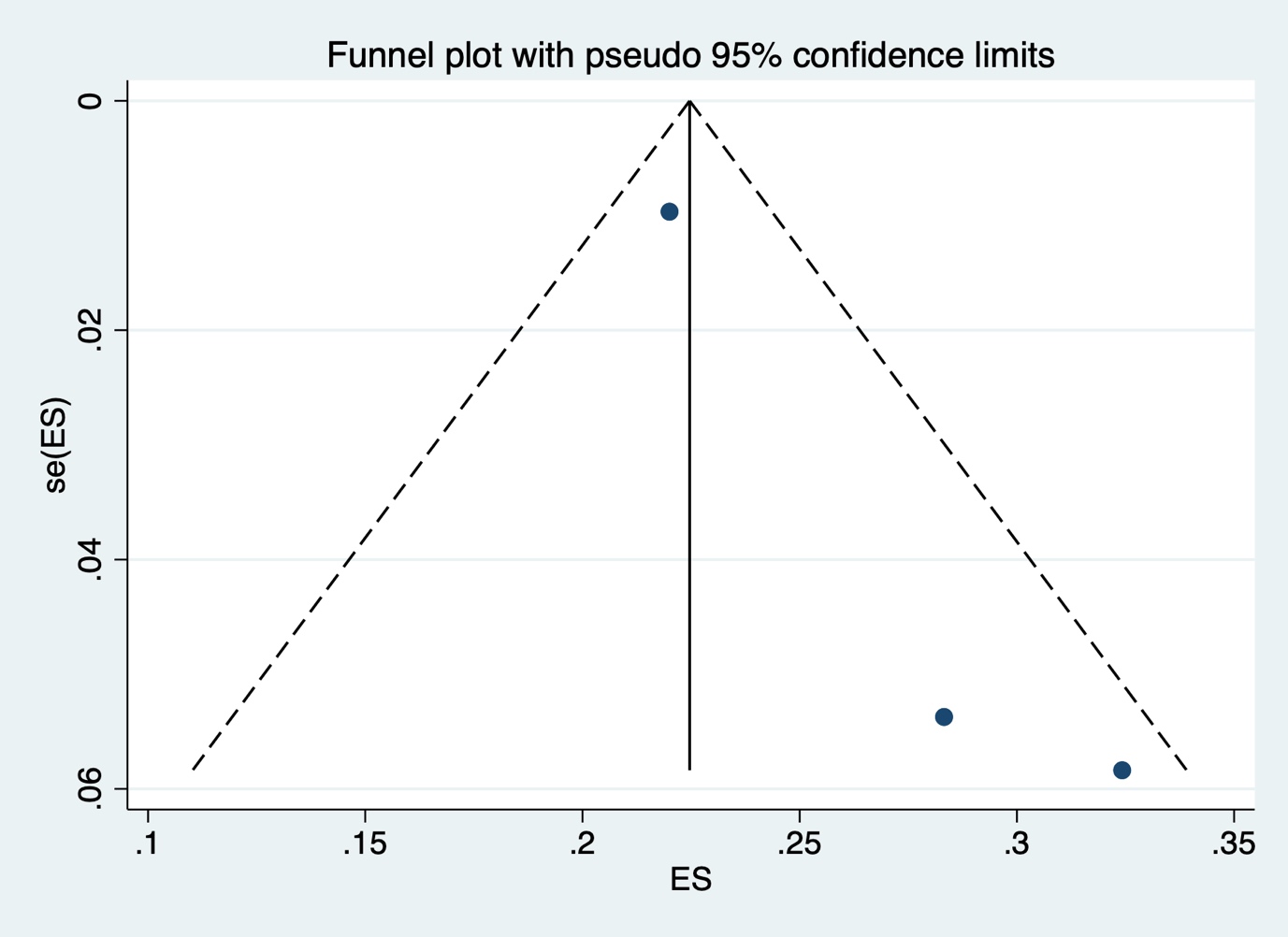
**

**Supplementary Figure 4 Funnel plot for people with STI symptoms**

**Egger p=0.269**

**
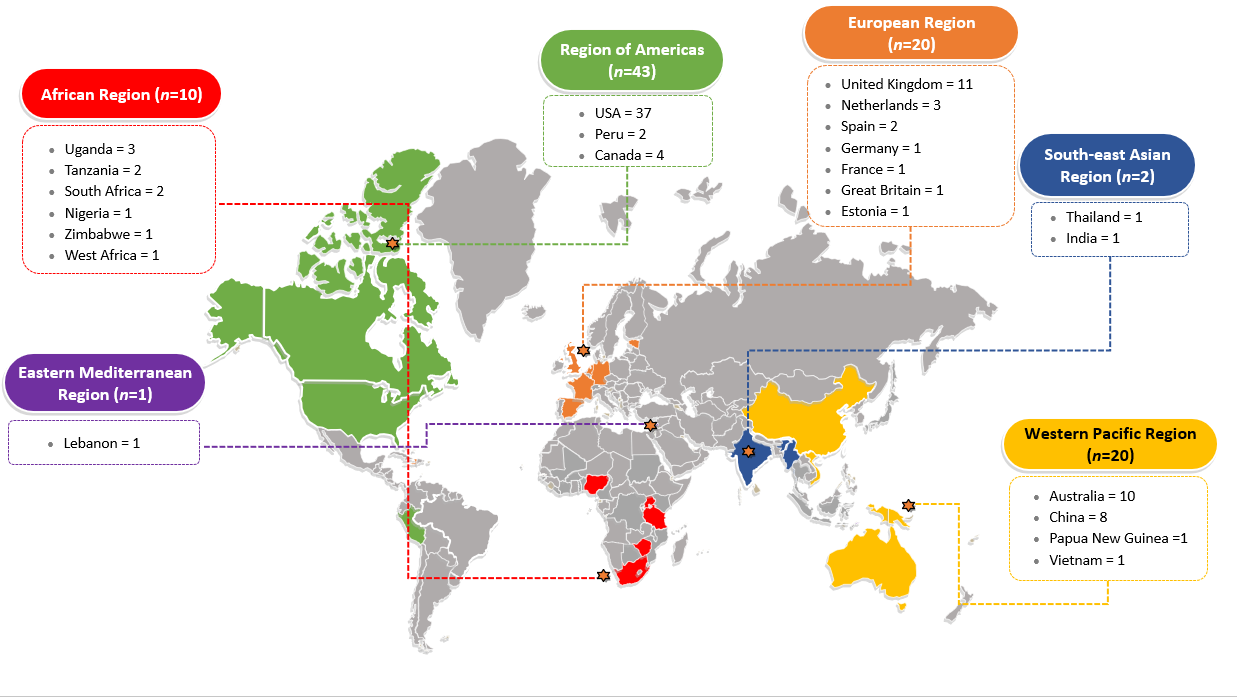
**

**Supplementary Figure 5 World map of included studies**
