# Supplementary material for: Missed opportunities for HIV testing among those who accessed sexually transmitted infection (STI) services, tested for STIs and diagnosed with STIs: a systematic review and meta‐analysis
Source: J Int AIDS Soc. 2023 Apr 26;26(4):e26049. doi: 10.1002/jia2.26049 (PMC10131090; doi:10.1002/jia2.26049)
Supplement: Supplementary file 2 — Supporting Information [file JIA2-26-e26049-s001.docx]

**Supplementary Table 1 Characteristics of included studies**

| Author Name | Year of study | Type of Study | Location (Country) | Study Population |
| --- | --- | --- | --- | --- |
| Adam[1] | 2011 | Observational | Australia | Sexual and gender minorities |
| Adekeye[2] | 2009 | Observational | USA | General |
| Assi[3] | 2015-2018 | Observational | Lebanon | Sexual and gender minorities |
| Avoundjian[4] | 2014-2016 | Observational | USA | Sexual and gender minorities |
| Badman[5] | 2014 | Observational | Papua New Guinea | Pregnant women |
| Baker U[6] | 2011-2014 | Observational,  Qualitative | Tanzania, Uganda | Pregnant women |
| Balan C [7] | 2016-2017 | Qualitative | USA | Sexual and gender minorities |
| Balira[8] | 2008-2009 | Observational | Tanzania | Healthcare workers & Pregnant women |
| Banerjee[9] | 2017 | Observational | UK | General |
| Barber[10] |  | Observational | Australia | Sexual and gender minorities |
| Barnes[11] | 2014-2015 | Observational | USA | General |
| Bauermeister[12] | 2013 | Observational | USA | Sexual and gender minorities |
| Bauermeister[13] |  | Experimental | USA | Sexual and gender minorities |
| Beck[14] |  | Modelling | USA | Sexual and gender minorities,  Youth |
| Bien[15] |  | Qualitative | China | Sexual and gender minorities |
| Bradley H[16] | 2009-2010 | Observational,  Qualitative | USA | General |
| Bremer[17] | 2010-2011 | Observational | Germany | Sex workers |
| Bristow[18] |  | Observational | Peru | Sexual and gender minorities |
| Brown L[19] | 2006 | Experimental | UK | General |
| Carcamo[20] | 2002 | Observational | Peru | General |
| Cayuelas [21] | 2013 | Observational | Spain | General |
| Chen J[22] | 2007 | Observational | USA | General |
| Chow E[23] | 2015-2016 | Observational | Australia | Sexual and gender minorities |
| Cushman[24] | 2014 | Observational | USA | Sexual and gender minorities |
| Fernandez[25] | 2008-2010 | Observational | Spain | General |
| Gamagedara[26] | 2009 | Observational | Australia | General |
| Gilbert, M.[27] | 2015-2016 | Observational | Canada | General |
| Golden[28] | 2010-2014 | Observational | USA | Sexual and gender minorities |
| Goulet[29] | 2010 | Observational | USA | Veterans |
| Goyal[30] | 2011 | Observational | USA | Youth |
| Goyal, M. K.[31] | 2011 | Observational | USA | Youth |
| Heard[32] |  | Observational | Australia | Sexual and gender minorities |
| Hottes[33] | 2011 | Qualitative | Canada | Sexual and gender minorities |
| Inghels[34] | 2017 | Observational | West Africa | General |
| Jichlinski[35] | 2010 to 2015 | Observational | USA | Youth |
| Jones[36] | 2013 | Qualitative | UK | Youth |
| Joore[37] | 2014 | Qualitative | Netherlands | General |
| Joore[38] | 2008-2013 | Observational | Netherlands | General |
| Joore[39] | 2009-2013 | Observational | Netherlands | General |
| Josten[40] | 2017 | Observational | USA | Youth providers |
| Kapadia[41] | 2010-2015 | Observational | USA | General |
| Kharsany[42] | 2005-2006 | Observational | South Africa | Women attending STI clinics |
| Katz[43] | 2012-2014 | Observational | USA | Sexual and gender minorities |
| Kilmarx[44] | 2014-2015 | Observational | Zimbabwe | General |
| Klein[45] | 2009-2011 | Observational | USA | General |
| Klein[46] | 2009 | Observational | USA | General |
| Knight[47] |  | Qualitative | Canada | Sexual and gender minorities |
| Lanier[48] | 2010-2011 | Observational,  Qualitative | USA | Physicians |
| Leon[49] | 2007 | Pragmatic cluster non-randomised controlled trial | South Africa | General |
| Li[50] | 2013-2014 | Observational |  | General |
| Llata[51] | 2010-2013 | Observational | USA | Sexual and gender minorities |
| Lopez[52] | 2010-2015 | Observational | USA | General |
| MacDonald[53] | 2004 | Observational | UK | General |
| Marsh[54] | 2011 | Observational | UK | Prisoners |
| Maxwell[55] |  | Observational | UK | General Practitioners |
| McDonagh[56] |  | Qualitative | UK | Sexual and gender minorities |
| Mohammed [57] | 2014 | Observational | UK | Black Africans  attending sexual health clinics |
| Moore[58] | 2014 | Observational | USA | Youth |
| Moore[59] | 2010-2011 | Observational | USA | Youth |
| Muhindo [60] | 2018 | Observational | Uganda | Sex workers |
| Muhindo [61] | 2019 | Observational | Uganda | Sex workers |
| Mullens[62] | 2016-2017 | Observational,  Qualitative | Australia | Sexual and gender minorities |
| Murtaugh[63] | 2013 | Observational | USA | General |
| Ngo[64] | 2007-2009 | Observational | Vietnam | Youth |
| Owusu-Edusei Jr[65] | 2012 | Observational | USA | Sexual and gender minorities |
| Owusu-Edusei Jr[66] |  | Modelling | China | Pregnant women |
| Pai[67] | 2008-2009 | Observational | India | Pregnant women |
| Petlo[68] | 2003-2009 | Observational | Australia | Sexual and gender minorities |
| Petsis[69] | 2014-2017 | Observational | USA | Youth |
| Phrasisombath[70] | 2010 | Qualitative | Thailand | Sex workers |
| Prabhu[71] | 2006-2008 | Modelling | USA | General  Sex workers |
| Rocchetti[72] | 2013 | Observational | France | General Practitioners |
| Ruutel[73] | 2012 | Observational | Estonia | People with indicator conditions |
| Saunders[74] | 2010 | Observational | Great Britain | Youth |
| Schechter[75] | 2011-2015 | Observational | USA | General |
| Scheim[76] | 2013 | Qualitative | Canada | Sexual and gender minorities |
| Selvey[77] | 2011-2015 | Observational | Australia | Sexual and gender minorities |
| Sharma[78] | 2017-2018 | Observational | USA | Sexual and gender minorities,  Youth |
| Slinkard[79] |  | Qualitative | USA | Older adults |
| Snow[80] | 2006-2007 | Observational | Australia | Sexual and gender minorities |
| Sullivan[81] |  | Observational | USA | Sexual and gender minorities |
| Tobin-West[82] | 2011 | Observational | Nigeria | General |
| Tucker[83] | 2009 | Observational | Guangdong China | Patients at STI Clinic |
| Tucker[84] | 2009 | Observational | South China | General |
| Tucker[85] | 2009-2010 | Observational | China | General |
| Underhill[86] | 2012-2014 | Qualitative | USA | Sexual and gender minorities |
| Wang[87] | 2018 | Observational | China | Sexual and gender minorities |
| Wang [88] | 2002 | Observational | China | General |
| Ward[89] | 2010-2014 | Observational | Australia | Aboriginal communities |
| Ward[89] | 2011 | Observational | Australia | Aboriginal communities |
| Waxman[90] | 2015 | Observational | USA | General |
| Williford[91] | 2015-2019 | Observational | USA | General |
| Wood[92] | 2013 | Observational | UK | Sexual and gender minorities |
| Youssef[93] | 2012 | Observational | UK, Brighton | General |
| Yumori[94] | 2019 | Observational | USA | General |
| Zhao[95] | 2016 | Observational | China | General |

**Supplementary Table 2 HIV positivity among people tested for HIV who attended an STI service, who were tested for STIs, who were diagnosed with STIs and people with STI symptoms.**

| Author | Recruitment Site | Population | HIV Positivity among people:  (n/N (%)) | | | |
| --- | --- | --- | --- | --- | --- | --- |
|  |  |  | Who attended a clinic with STI testing services. | Who were tested for STIs | Who were diagnosed with STIs | with STI symptoms |
| Assi A, 2019[3] | STI Clinic | MSM | 119/2126 (5.59) |  |  |  |
| Banerjee, 2020[9] | STI Clinic | Mixed | 42/16229 (0.25) |  |  |  |
| Bremer, 2016[17] | STI Clinic | Sex workers | 8/3882 (0.20) |  |  |  |
| Chow, 2018[23] | STI Clinic | MSM | 7/3400 (0.20) |  |  |  |
| Kharsany, 2010[42] | STI Clinic | Women attending STI clinics | 1378/2439 (56.49) |  |  |  |
| Katz, 2016[43] | STI Clinic | MSM | 104/4441 (2.34) |  |  |  |
| Kilmarx, P. H, 2018[44] | STI Clinic | Mixed | 201/489 (41.10) |  |  |  |
| Leon, N, 2010[49] | STI Clinic | Mixed | 326/1752 (18.60) |  |  |  |
| Leon, N, 2010[49] | STI Clinic | Mixed | 605/2821 (21.44) |  |  |  |
| Llata, 2018[51] | STI Clinic | MSM | 640/38915 (1.64) |  |  |  |
| Selvey, 2018[77] | STI Clinic | MSM | 46/2753 (1.67) |  |  |  |
| Zhao, P.[95] | STI Clinic | Mixed | 26/1177 (2.20) |  |  |  |
| Balira, 2015[8] | Hospital  Reproductive and child health clinics | Health workers  Pregnant women |  | 0/903  (0) |  |  |
| Carcamo, 2012[20] | Outpatient | MSM |  | 22/5681 (0.38) |  |  |
| Carcamo, 2012[20] | Outpatient | MSM |  | 3/5799 (0.05) |  |  |
| Kapadia, S. N, 2018[97] | Hospital  Outpatient  Emergency Department | Male |  | 31/11515 (0.26) |  |  |
| Kapadia, S. N, 2018[97] | Hospital  Outpatient  Emergency Department | Female |  | 6/56138 (0.01) |  |  |
| Wood, 2014[92] | Other - Suana | MSM |  | 1/30 (3.33) |  |  |
| Wood, 2014[92] | Other – Postal kits were sent | MSM |  | 0/30 (0) |  |  |
| Wood, 2014[92] | STI Clinic | MSM |  | 1/30 (3.33) |  |  |
| Golden, 2015[28] | Other- partner service interviewed by public health staff | MSM |  |  | 165/4631 (3.56) |  |
| Cayuelas, 2019[21] | Community-based facility/GP | Mixed |  |  | 2/34  (5.88) |  |
| Cayuelas, 2019[21] | Community-based facility/GP | Mixed |  |  | 4/87  (4.59) |  |
| Cayuelas, 2019[21] | Community-based facility/GP | Mixed |  |  | 3/73  (4.10) |  |
| Li, 2016[98] | STI Clinic | Mixed |  |  | 63/2668  (2.36) |  |
| Petsis, 2020[69] | Community based facility/GP | Youth |  |  | 1/1001  (1) |  |
| Williford, 2021[91] | STI Clinic  Emergency Department | Mixed |  |  | 15/961  (1.56) |  |
| Schechter, S. B, 2017[75] | Emergency Department | Males |  |  |  | 0/95  (0) |

**REFERENCES**

1. Adam P, Wit J, Bourne C, Knox D, Purchas J. Promoting Regular Testing: An Examination of HIV and STI Testing Routines and Associated Socio-Demographic, Behavioral and Social-Cognitive Factors Among Men Who have Sex with Men in New South Wales, Australia. AIDS & Behavior. 2014;18(5):921-32.

2. Adekeye OA, Abara WE, Xu J, Lee JM, Rust G, Satcher D. HIV Screening Rates among Medicaid Enrollees Diagnosed with Other Sexually Transmitted Infections. PLoS One. 2016;11(8):e0161560-e.

3. Assi A, Abu Zaki S, Ghosn J, Kinge N, Naous J, Ghanem A, et al. Prevalence of HIV and other sexually transmitted infections and their association with sexual practices and substance use among 2238 MSM in Lebanon. Scientific Reports.9(1):15142.

4. Avoundjian T, Stewart J, Peyton D, Lewis C, Johnson K, Glick SN, et al. Integrating HIV testing into syphilis partner services in Mississippi to improve HIV case finding. Sexually Transmitted Diseases. 2018;03.

5. Badman SG, Vallely LM, Toliman P, Kariwiga G, Lote B, Pomat W, et al. A novel point-of-care testing strategy for sexually transmitted infections among pregnant women in high-burden settings: results of a feasibility study in Papua New Guinea. BMC Infectious Diseases.16:250.

6. Baker U, Okuga M, Waiswa P, Manzi F, Peterson S, Hanson C, et al. Bottlenecks in the implementation of essential screening tests in antenatal care: Syphilis, HIV, and anemia testing in rural Tanzania and Uganda. International Journal of Gynaecology & Obstetrics.130 Suppl 1:S43-50.

7. Balan IC, Lopez-Rios J, Nayak S, Lentz C, Arumugam S, Kutner B, et al. SMARTtest: A Smartphone App to Facilitate HIV and Syphilis Self- and Partner-Testing, Interpretation of Results, and Linkage to Care. AIDS & Behavior.24(5):1560-73.

8. Balira R, Mabey D, Weiss H, Ross DA, Changalucha J, Watson-Jones D. The need for further integration of services to prevent mother-to-child transmission of HIV and syphilis in Mwanza City, Tanzania. (Special Issue: Accelerating dual elimination of mother-to-child transmission of syphilis and HIV through use of new diagnostic tools.). International Journal of Gynecology & Obstetrics. 2015;130(Suppl. 1):S51-S7.

9. Banerjee P, Madhwapathi V, Thorley N, Radcliffe K. A service evaluation comparing home-based testing to clinic-based testing for HIV, syphilis and hepatitis B in Birmingham and Solihull. International Journal of STD & AIDS.31(7):613-8.

10. Barber B, Hellard M, Jenkinson R, Spelman T, Stoove M. Sexual history taking and sexually transmissible infection screening practices among men who have sex with men: a survey of Victorian general practitioners. Sexual Health.8(3):349-54.

11. Barnes A, Jetelina KK, Betts AC, Mendoza T, Pranavi S, Tiro JA. Emergency department testing patterns for sexually transmitted diseases in North Texas. Sexually Transmitted Diseases. 2019;46(7):434-9.

12. Bauermeister J, Pingel E, Jadwin-Cakmak L, Meanley S, Alapati D, Moore M, et al. The Use of Mystery Shopping for Quality Assurance Evaluations of HIV/STI Testing Sites Offering Services to Young Gay and Bisexual Men. AIDS & Behavior. 2015;19(10):1919-27.

13. Bauermeister J, Pingel E, Jadwin-Cakmak L, Harper G, Horvath K, Weiss G, et al. Acceptability and Preliminary Efficacy of a Tailored Online HIV/STI Testing Intervention for Young Men who have Sex with Men: The Get Connected! Program. AIDS & Behavior. 2015;19(10):1860-74.

14. Beck E, Armbruster B, Birkett M, Mustanski B. The value of timely implementation in HIV/STI testing: Cost-effectiveness vs. Speed of intervention rollout. Value in Health. 2016;19 (7):A365.

15. Bien CH, Muessig KE, Lee R, Lo EJ, Yang L, Yang B, et al. HIV and syphilis testing preferences among men who have sex with men in South China: a qualitative analysis to inform sexual health services. Plos One. 2015;10(4).

16. Bradley H, Asbel L, Bernstein K, Mattson M, Pathela P, Mohamed M, et al. HIV Testing Among Patients Infected with Neisseria gonorrhoeae: STD Surveillance Network, United States, 2009-2010. AIDS & Behavior. 2013;17(3):1205-10.

17. Bremer V, Haar K, Gassowski M, Hamouda O, Nielsen S. STI tests and proportion of positive tests in female sex workers attending local public health departments in Germany in 2010/11. BMC Public Health.16(1):1175.

18. Bristow CC, Kojima N, Lee S, Leon SR, Ramos LB, Konda KA, et al. HIV and syphilis testing preferences among men who have sex with men and among transgender women in Lima, Peru. Plos One. 2018;13(10).

19. Brown L, Patel S, Ives NJ, McDermott C, Ross JD. Is non-invasive testing for sexually transmitted infections an efficient and acceptable alternative for patients? A randomised controlled trial. Sexually Transmitted Infections.86(7):525-31.

20. Cárcamo CP, Campos PE, García PJ, Hughes JP, Garnett GP, Holmes KK, et al. Prevalences of sexually transmitted infections in young adults and female sex workers in Peru: a national population-based survey. Lancet Infectious Diseases. 2012;12(10):765-73.

21. Cayuelas Redondo L, Ruiz M, Kostov B, Sequeira E, Noguera P, Herrero MA, et al. Indicator condition-guided HIV testing with an electronic prompt in primary healthcare: a before and after evaluation of an intervention. Sexually Transmitted Infections. 2019;95(4):238-43.

22. Chen JY, Ma Q, Everhard F, Yermilov I, Tian H, Mayer KH. HIV screening in commercially insured patients screened or diagnosed with sexually transmitted diseases or blood-borne pathogens. Sexually Transmitted Diseases.38(6):522-7.

23. Chow EPF, Fortune R, Dobinson S, Wakefield T, Read TRH, Chen MY, et al. Evaluation of the Implementation of a New Nurse-Led Express "Test-And-Go" Human Immunodeficiency Virus/Sexually Transmitted Infection Testing Service for Men Who Have Sex With Men at a Sexual Health Center in Melbourne, Australia. Sexually Transmitted Diseases.45(6):429-34.

24. Cushman T, Graves SK, Little S. Attitudes and preferences regarding the use of point-of-care and near-care sexually transmitted disease (STD) and human immunodeficiency virus (HIV) tests in San Diego area men who have sex with men. Open Forum Infectious Diseases Conference: ID Week. 2016;3(Supplement 1).

25. Fernandez-Balbuena S, Hoyos J, Rosales-Statkus ME, Nardone A, Vallejo F, Ruiz M, et al. Low HIV testing uptake following diagnosis of a sexually transmitted infection in Spain: implications for the implementation of efficient strategies to reduce the undiagnosed HIV epidemic. AIDS Care. 2016;28(6):677-83.

26. Gamagedara N, Dobinson S, Cummings R, Fairley CK, Lee D. An evaluation of an express testing service for sexually transmissible infections in low-risk clients without complications. Sexual Health.11(1):37-41.

27. Gilbert M, Thomson K, Salway T, Haag D, Grennan T, Fairley CK, et al. Differences in experiences of barriers to STI testing between clients of the internet-based diagnostic testing service GetCheckedOnline.com and an STI clinic in Vancouver, Canada. Sexually Transmitted Infections.95(2):151-6.

28. Golden MR, Katz DA, Kern D, Heal D, Kerani R, Dombrowski JC. Sexually transmitted disease partner services increase HIV testing among men who have sex with men. Topics in Antiviral Medicine. 2015;23 (E-1):507.

29. Goulet JL, Martinello RA, Bathulapalli H, Higgins D, Driscoll MA, Brandt CA, et al. STI diagnosis and HIV testing among OEF/OIF/OND veterans. Medical Care.52(12):1064-7.

30. Goyal M, Witt R, Gerber J, Hayes K, Zaoutis T. Physician adherence to sexual health and STI/HIV screening recommendations during routine adolescent health visits. Journal of Adolescent Health. 2013;52(2):S84.

31. Goyal MK, Witt R, Hayes KL, Zaoutis TE, Gerber JS. Clinician adherence to recommendations for screening of adolescents for sexual activity and sexually transmitted infection/human immunodeficiency virus. Journal of Pediatrics.165(2):343-7.

32. Heard E, Oost E, McDaid L, Mutch A, Dean J, Fitzgerald L. How can HIV/STI testing services be more accessible and acceptable for gender and sexually diverse young people? A brief report exploring young people's perspectives in Queensland. Health Promotion Journal of Australia.31(1):150-5.

33. Hottes TS, Farrell J, Bondyra M, Haag D, Shoveller J, Gilbert M. Internet-based HIV and sexually transmitted infection testing in British Columbia, Canada: opinions and expectations of prospective clients. Journal of Medical Internet Research.14(2):e41.

34. Inghels M, Kouassi AK, Niangoran S, Bekelynck A, Carillon S, Sika L, et al. Cascade of Provider-Initiated Human Immunodeficiency Virus Testing and Counselling at Specific Life Events (Pregnancy, Sexually Transmitted Infections, Marriage) in Cote d'Ivoire. Sexually Transmitted Diseases. 2020;47(1):54-61.

35. Jichlinski A, Badolato GM, Pastor W, Goyal MK. 250 HIV and Syphilis Testing and Antibiotic Administration in Adolescents Diagnosed With Pelvic Inflammatory Disease in Pediatric Emergency Departments. Annals of Emergency Medicine. 2017;70:S99-S.

36. Jones LF, Ricketts E, Town K, Rugman C, Lecky D, Folkard K, et al. Chlamydia and HIV testing, contraception advice, and free condoms offered in general practice: a qualitative interview study of young adults' perceptions of this initiative. British Journal of General Practice.67(660):e490-e500.

37. Joore IK, Roosmalen SLv, Bergen JEAMv, Dijk Nv. General practitioners' barriers and facilitators towards new provider-initiated HIV testing strategies: a qualitative study. International Journal of STD & AIDS. 2017;28(5):459-66.

38. Joore IK, Reukers DF, Donker GA, van Sighem AI, Op de Coul EL, Prins JM, et al. Missed opportunities to offer HIV tests to high-risk groups during general practitioners' STI-related consultations: an observational study. BMJ Open.6(1):e009194.

39. Joore IK, Twisk DE, Vanrolleghem AM, de Ridder M, Geerlings SE, van Bergen JEAM, et al. The need to scale up HIV indicator condition-guided testing for early case-finding: a case-control study in primary care. BMC Fam Pract. 2016;17(1):161-.

40. Josten MS, Keeshin S. Knowledge, practices, and attitudes of youth providers about STI, HIV testing, and prep. Open Forum Infectious Diseases. 2018;5 (Supplement 1):S697.

41. Kapadia S, Vaamonde C, Singh H, Jones S, Merrick S. Missed opportunities for human immunodeficiency virus (HIV) testing at a large urban healthcare system from 2010 to 2015. Open Forum Infectious Diseases Conference: ID Week. 2016;3(Supplement 1).

42. Kharsany AB, Karim QA, Karim SS. Uptake of provider-initiated HIV testing and counseling among women attending an urban sexually transmitted disease clinic in South Africa - missed opportunities for early diagnosis of HIV infection. AIDS Care.22(5):533-7.

43. Katz DA, Dombrowski JC, Kerani RP, Aubin MR, Kern DA, Heal DD, et al. Integrating HIV Testing as an Outcome of STD Partner Services for Men Who Have Sex with Men. AIDS Patient Care & Stds.30(5):208-14.

44. Kilmarx PH, Gonese E, Lewis DA, Chirenje ZM, Barr BAT, Latif AS, et al. HIV infection in patients with sexually transmitted infections in Zimbabwe - Results from the Zimbabwe STI etiology study. PLoS ONE [Electronic Resource].13(6):e0198683.

45. Klein P, Bishop A, Leone P. Hiv testing of patients receiving an std evaluation in a north carolina community health center. Sexually Transmitted Infections. 2011;87:A208.

46. Klein PW, Martin IBK, Quinlivan EB, Gay CL, Leone PA. Missed opportunities for concurrent HIV-STD testing in an academic emergency department. (Special Issue: Program collaboration and service integration in the prevention and control of HIV infection, viral hepatitis, STDs, and tuberculosis in the U.S.: lessons learned from the field.). Public Health Reports. 2014;129(1 (Suppl.1):12-20.

47. Knight R, Shoveller JA, Oliffe JL, Gilbert M, Goldenberg S. Heteronormativity hurts everyone: experiences of young men and clinicians with sexually transmitted infection/HIV testing in British Columbia, Canada. Health: an Interdisciplinary Journal for the Social Study of Health, Illness & Medicine.17(5):441-59.

48. Lanier Y, Castellanos T, Barrow RY, Jordan WC, Caine V, Sutton MY. Brief sexual histories and routine HIV/STD testing by medical providers. AIDS Patient Care & Stds.28(3):113-20.

49. Leon N, Naidoo P, Mathews C, Lewin S, Lombard C. The impact of provider-initiated (opt-out) HIV testing and counseling of patients with sexually transmitted infection in Cape Town, South Africa: a controlled trial. Implementation Science.5:8.

50. Li J, Jiang N, Yue X, Gong X. [HIV detection and prevalence among sexullay transmitted diseases clinic patients in seven provinces (Autonomous Region)]. Chung-Hua Liu Hsing Ping Hsueh Tsa Chih Chinese Journal of Epidemiology.37(3):358-61.

51. Llata E, Braxton J, Asbel L, Kerani RP, Murphy R, Pugsley R, et al. New Human Immunodeficiency Virus Diagnoses Among Men Who Have Sex With Men Attending Sexually Transmitted Disease Clinics, STD Surveillance Network, January 2010 to June 2013. Sexually Transmitted Diseases.45(9):577-82.

52. Lopez R, Hustey FM, Schold JD, Seballos SS, Phelan MP. 243 Emergency Department Sexually Transmitted Infection Testing and Compliance With CDC HIV Testing Guidelines in a National Sample of Emergency Departments. Annals of Emergency Medicine. 2019;74 (4 Supplement):S95.

53. MacDonald R, Goodall L, Nair V, Baguley S, Clutterbuck D. Completion of a British Association for Sexual Health and HIV regional audit loop: HIV testing in genitourinary medicine clinics in Scotland in 2004 and 2008. International Journal of STD & AIDS.21(9):648-9.

54. Marsh K, Chan S, Wheatley N, Duffell S, Lau R, Hughes G. Missed STI and HIV testing opportunities among male prisoners in England. Sexually Transmitted Infections Conference: STI and AIDS World Congress. 2013;89(SUPPL. 1).

55. Maxwell S. General Practitioners' views and experiences on the barriers and facilitators that men who have sex with men have when accessing primary care for HIV testing and sexual health screening. Primary Health Care Research & Development.19(2):205-9.

56. McDonagh L, Omran L, Curtis T, Pach S, Saunders J, Cassell J, et al. 'you feel invisible': A qualitative exploration of young LGBT+ people's attitudes towards STI/HIV testing in primary care. Sexually Transmitted Infections. 2019;95 (Supplement 1):A63.

57. Mohammed H, Dabrera G, Furegato M, Yin Z, Nardone A, Hughes G. Refusal of HIV testing among black Africans attending sexual health clinics in England, 2014: a review of surveillance data. Sexually Transmitted Infections.93(3):217-20.

58. Moore MJ, Barr E, Wilson K, Griner S. Support for Offering Sexual Health Services Through School-Based Health Clinics. Journal of School Health.86(9):660-8.

59. Moore EW. Human immunodeficiency virus and chlamydia/gonorrhea testing among heterosexual college students: who is getting tested and why do some not? Journal of American College Health.61(4):196-202.

60. Muhindo R, Castelnuovo B, Mujugira A, Parkes-Ratanshi R, Sewankambo NK, Kiguli J, et al. Psychosocial correlates of regular syphilis and HIV screening practices among female sex workers in Uganda: a cross-sectional survey. AIDS Research & Therapy [Electronic Resource].16(1):28.

61. Muhindo R, Mujugira A, Castelnuovo B, Sewankambo NK, Parkes-Ratanshi R, Kiguli J, et al. HIV and syphilis testing behaviors among heterosexual male and female sex workers in Uganda. AIDS Research & Therapy [Electronic Resource].17(1):48.

62. Mullens AB, Duyker J, Brownlow C, Lemoire J, Daken K, Gow J. Point-of-care testing (POCT) for HIV/STI targeting MSM in regional Australia at community 'beat' locations. BMC Health Services Research.19(1):93.

63. Murtaugh KL, Leibowitz A, Chen X, Pourat N. Missed opportunities for HIV screening of new enrollees in California's low income health program. Aids Education and Prevention. 2020;32(1):25-35.

64. Ngo AD, Ha TH, Rule J, Dang CV. Peer-based education and the integration of HIV and sexual and reproductive health services for young people in Vietnam: evidence from a project evaluation. Plos One. 2013;8(11).

65. Owusu-Edusei Jr K, Gift TL, Patton ME, Johnson DB, Valentine JA, Owusu-Edusei K, Jr. Estimating the Total Annual Direct Cost of Providing Sexually Transmitted Infection and HIV Testing and Counseling for Men Who Have Sex With Men in the United States. Sexually Transmitted Diseases. 2015;42(10):586-9.

66. Owusu-Edusei Jr K, Tao G, Gift TL, Wang A, Wang L, Tun Y, et al. Cost-effectiveness of Integrated Routine Offering of Prenatal HIV and Syphilis Screening in China. Sexually Transmitted Diseases. 2014;41(2):103-10.

67. Pai NP, Kurji J, Singam A, Barick R, Jafari Y, Klein MB, et al. Simultaneous triple point-of-care testing for HIV, syphilis and hepatitis B virus to prevent mother-to-child transmission in India. International Journal of STD & AIDS.23(5):319-24.

68. Petlo T, Fairley CK, Whitton B, Coles K, Chen MY. HIV-testing of men who have sex with men: variable testing rates among clinicians. (Special Issue: HIV testing.). International Journal of STD & AIDS. 2011;22(12):727-9.

69. Petsis D, Jungwon M, Yuan-Shung VH, Akers AY, Wood S. HIV Testing Among Adolescents With Acute Sexually Transmitted Infections. Pediatrics. 2020;145(4):1-8.

70. Phrasisombath K, Thomsen S, Sychareun V, Faxelid E. Care seeking behaviour and barriers to accessing services for sexually transmitted infections among female sex workers in Laos: a cross-sectional study. BMC Health Services Research. 2012;12.

71. Prabhu VS, Farnham PG, Hutchinson AB, Soorapanth S, Heffelfinger JD, Golden MR, et al. Cost-effectiveness of HIV screening in STD clinics, emergency departments, and inpatient units: a model-based analysis. PLoS ONE [Electronic Resource].6(5):e19936.

72. Rocchetti V, Viard JP. Family practitioners screening for HIV infection. Medecine et Maladies Infectieuses.45(5):157-64.

73. Ruutel K, Lemsalu L, Latt S, Opt TbH. Monitoring HIV-indicator condition guided HIV testing in Estonia. HIV Medicine.19 Suppl 1:47-51.

74. Saunders JM, Mercer CH, Sutcliffe LJ, Hart GJ, Cassell J, Estcourt CS. Where do young men want to access STI screening? A stratified random probability sample survey of young men in Great Britain. Sexually Transmitted Infections.88(6):427-32.

75. Schechter SB, Romo DL, Cohall AT, Neu NM. Approach to Human Immunodeficiency Virus/Sexually Transmitted Infection Testing for Men at an Urban Urgent Care Center. Sexually Transmitted Diseases.44(4):255-9.

76. Scheim AI, Travers R. Barriers and facilitators to HIV and sexually transmitted infections testing for gay, bisexual, and other transgender men who have sex with men. AIDS Care.29(8):990-5.

77. Selvey LA, Slimings C, Adams E, Manuel J. Incidence and predictors of HIV, chlamydia and gonorrhoea among men who have sex with men attending a peer-based clinic. Sexual Health. 2018;15(5):451-9.

78. Sharma A, Kahle E, Todd K, Peitzmeier S, Stephenson R. Variations in Testing for HIV and Other Sexually Transmitted Infections Across Gender Identity Among Transgender Youth. Transgender Health.4(1):46-57.

79. Slinkard MS, Kazer MW. Older adults and HIV and STI screening: the patient perspective. Geriatric Nursing.32(5):341-9.

80. Snow AF, Cummings R, Owen L, El-Hyak C, Hellard ME, Vodstrcil L, et al. Introduction of a sexual health practice nurse increases sti testing among MSM in general practice. Sexually Transmitted Infections. 2011;87:A98.

81. Sullivan SP, Sullivan PS, Stephenson R. Acceptability and Feasibility of a Telehealth Intervention for STI Testing Among Male Couples. Aids and Behavior.

82. Tobin-West CI, Lawson AM. Stigma and underutilization of facility-based sexually transmitted infection services undermine human immunodeficiency virus testing in rural communities of Rivers State, Nigeria. International Journal of Health and Allied Sciences. 2013;2(2):108-14.

83. Tucker JD, Yang L, Yang B, Young D, Henderson GE, Huang S, et al. Prior HIV testing among STD patients in Guangdong Province, China: opportunities for expanding detection of sexually transmitted HIV infection. Sexually Transmitted Diseases. 2012;39(3):182-7.

84. Tucker JD, Walensky RP, Yang L-G, Yang B, Bangsberg DR, Chen X-S, et al. Expanding provider-initiated HIV testing at STI clinics in China. AIDS Care. 2012;24(10):1316-9.

85. Tucker JD, Yang L, Yang B, Zheng H, Chang H, Wang C, et al. A twin response to twin epidemics: integrated HIV/syphilis testing at STI clinics in South China. JAIDS, Journal of Acquired Immune Deficiency Syndromes. 2011;57(5):e106-e11.

86. Underhill K, Morrow KM, Colleran CM, Holcomb R, Operario D, Calabrese SK, et al. Access to healthcare, HIV/STI testing, and preferred pre-exposure prophylaxis providers among men who have sex with men and men who engage in street-based sex work in the US. PLoS ONE [Electronic Resource].9(11):e112425.

87. Wang C, Cheng W, Li C, Tang W, Ong JJ, Smith MK, et al. Syphilis self-testing: a nationwide pragmatic study among men who have sex with men in China. Clinical Infectious Diseases. 2020;70(10):2178-86.

88. Wang B, Li X, Stanton B, McGuire J. Correlates of HIV/STD testing and willingness to test among rural-to-urban migrants in China. AIDS & Behavior.14(4):891-903.

89. Ward JS, Dyda A, McGregor S, Rumbold A, Garton L, Donovan B, et al. Low HIV testing rates among people with a sexually transmissible infection diagnosis in remote Aboriginal communities. Medical Journal of Australia.205(4):168-71.

90. Waxman M, Ata A, Frisch A, Sutton L. Rates of emergency department human immunodeficiency virus (HIV) tested in patients tested for sexually transmitted diseases. Open Forum Infectious Diseases Conference: ID Week. 2016;3(Supplement 1).

91. Williford SL, Humes E, Greenbaum A, Schumacher CM. HIV Screening Among Gonorrhea-Diagnosed Individuals; Baltimore, Maryland; April 2015 to April 2019. Sexually Transmitted Diseases.48(1):42-8.

92. Wood M, Ellks R, Grobicki M. Outreach sexual infection screening and postal tests in men who have sex with men: are they comparable to clinic screening? International Journal of STD & AIDS.26(6):428-31.

93. Youssef E, Sanghera T, Bexley A, Hayes M, Perry N, Dosekun O, et al. HIV testing in patients presenting with indicator conditions in outpatient settings: offer and uptake rates, and educational and active interventions. International Journal of STD & AIDS. 2018;29(13):1289-94.

94. Yumori C, Zucker J, Theodore D, Chang M, Carnevale C, Slowikowski J, et al. Women Are Less Likely to Be Tested for HIV or Offered Preexposure Prophylaxis at the Time of Sexually Transmitted Infection Diagnosis. Sexually Transmitted Diseases.48(1):32-6.

95. Zhao P, Tang W, Cheng H, Huang S, Zheng H, Yang B, et al. Uptake of provider-initiated HIV and syphilis testing among heterosexual STD clinic patients in Guangdong, China: results from a cross-sectional study. BMJ Open. 2020;10(12):e041503.

96. Tucker JD, Yang LG, Yang B, Young D, Henderson GE, Huang SJ, et al. Prior HIV testing among STD patients in Guangdong Province, China: Opportunities for expanding detection of sexually transmitted HIV infection. []. Sexually Transmitted Diseases. 2011;24.

97. Kapadia SN, Singh HK, Jones S, Merrick S, Vaamonde CM. Missed Opportunities for HIV Testing of Patients Tested for Sexually Transmitted Infections at a Large Urban Health Care System From 2010 to 2015. Open Forum Infectious Diseases.5(7):ofy165.

98. Li J, Jiang N, Yue X, Gong X. HIV detection and prevalence among sexullay transmitted diseases clinic patients in seven provinces (Autonomous Region). [Chinese]. Zhonghua liu xing bing xue za zhi = Zhonghua liuxingbingxue zazhi. 2016;37(3):358-61.
