# Supplementary material for: Missed opportunities for HIV testing among those who accessed sexually transmitted infection (STI) services, tested for STIs and diagnosed with STIs: a systematic review and meta‐analysis
Source: J Int AIDS Soc. 2023 Apr 26;26(4):e26049. doi: 10.1002/jia2.26049 (PMC10131090; doi:10.1002/jia2.26049)
Supplement: Supplementary file 4 — Supporting Information [file JIA2-26-e26049-s004.docx]

**CHEERS Checklist- Modelling Studies**

|  | **Beck, 2016[1]** | **Owusu-Edusei, 2014[2]** | **Prabhu, 2011[3]** |
| --- | --- | --- | --- |
| Title | 1 | 1 | 1 |
| Abstract | 1 | 1 | 1 |
| Background and objectives | 0 | 1 | 1 |
| Target population and subgroups | 1 | 1 | 1 |
| Setting and location | 1 | 1 | 1 |
| Study perspective | 0 | 1 | 1 |
| Comparators | 1 | 1 | 1 |
| Time horizon | 1 | 0 | 0 |
| Discount rate | 0 | 1 | 1 |
| Choice of health outcomes | 1 | 1 | 1 |
| Measurement of effectiveness | 0 | 1 | 1 |
| Measurement and valuation of preference-based outcomes | 0 | 1 | 1 |
| Estimating resources and costs | 0 | 1 | 1 |
| Currency, price date, and conversion | 0 | 1 | 1 |
| Choice of model | 1 | 1 | 1 |
| Assumptions | 1 | 1 | 1 |
| Analytical methods | 0 | 1 | 1 |
| Study parameters | 0 | 1 | 1 |
| Incremental costs and outcomes | 1 | 1 | 1 |
| Characterising uncertainty | 0 | 1 | 1 |
| Characterising heterogeneity | 0 | 1 | 1 |
| Study findings, limitations,generalisability, and  current knowledge | 0 | 1 | 1 |
| Source of funding | 0 | 1 | 1 |
| Conflicts of interest | 0 | 1 | 1 |
| **Total score (Out of 24)** | 9 | 20 | 21 |

ROB 2.0 – RCT

| **Authors** | **Bauermeister, 2015[4]** | **Brown, 2010[5]** | **Sharma, 2019[6]** |
| --- | --- | --- | --- |
| Domain 1: Risk of bias arising from the randomization process | | | |
| Was the allocation sequence random? | Y | Y | NI |
| Was the allocation sequence concealed until participants were enrolled and assigned to interventions? | Y | Y | Y |
| Did baseline differences between intervention groups suggest a problem with the randomization process? | N | N | N |
| Risk-of-bias judgement | Low risk | Low risk | Some concerns |
| Domain 2: Risk of bias due to deviations from the intended interventions (effect of assignment to intervention) | | | |
| Were participants aware of their assigned intervention during the trial? | N | N | N |
| Were carers and people delivering the interventions aware of participants' assigned intervention during the trial? | N | N | N |
| If Y/PY/NI to 2.1 or 2.2: Were there deviations from the intended intervention that arose because of the trial context? | NA | NA | NA |
| If Y/PY to 2.3: Were these deviations likely to have affected the outcome? | NA | NA | NA |
| If Y/PY/NI to 2.4: Were these deviations from intended intervention balanced between groups? | NA | NA | NA |
| Was an appropriate analysis used to estimate the effect of assignment to intervention? | Y | Y | Y |
| If N/PN/NI to 2.6: Was there potential for a substantial impact (on the result) of the failure to analyse participants in the group to which they were randomized? | NA | NA | NA |
| Risk-of-bias judgement | Low risk | Low risk | Low risk |
| Domain 3: Risk of bias due to missing outcome data | | | |
| Were data for this outcome available for all, or nearly all, participants randomized? | Y | Y | Y |
| If N/PN/NI to 3.1: Is there evidence that the result was not biased by missing outcome data? | NA | NA | NA |
| If N/PN to 3.2: Could missingness in the outcome depend on its true value? | NA | NA | NA |
| If Y/PY/NI to 3.3: Is it likely that missingness in the outcome depended on its true value? | NA | NA | NA |
| Risk-of-bias judgement | Low risk | Low risk | Low risk |
| Domain 4: Risk of bias in measurement of the outcome | | | |
| Was the method of measuring the outcome inappropriate? | N | N | N |
| Could measurement or ascertainment of the outcome have differed between intervention groups? | N | N | N |
| If N/PN/NI to 4.1 and 4.2: Were outcome assessors aware of the intervention received by study participants? | N | N | N |
| If Y/PY/NI to 4.3: Could assessment of the outcome have been influenced by knowledge of intervention received? | NA | NA | NA |
| If Y/PY/NI to 4.4: Is it likely that assessment of the outcome was influenced by knowledge of intervention received? | NA | NA | NA |
| Risk-of-bias judgement | Low risk | Low risk | Low risk |
| Domain 5: Risk of bias in selection of the reported result | | | |
| Were the data that produced this result analysed in accordance with a pre-specified analysis plan that was finalized before unblinded outcome data were available for analysis? | Y | Y | Y |
| Is the numerical result being assessed likely to have been selected, on the basis of the results, from multiple eligible outcome measurements (e.g., scales, definitions, time points) within the outcome domain? | N | N | N |
| Is the numerical result being assessed likely to have been selected, on the basis of the results, from multiple eligible analyses of the data? | N | N | N |
| Risk-of-bias judgement | Low risk | Low risk | Low risk |
| Overall risk of bias (Low / High / Some concerns) | **Low risk** | **Low risk** | **Some concerns** |

**Newcastle-Ottawa Scale - Cohort Studies**

| **Authors** | Representativeness of the exposed cohort  a) truly representative of the average _______________ (describe) in the community    b) somewhat representative of the average ______________ in the community  c) selected group of users eg nurses, volunteers  d) no description of the derivation of the cohort | Selection of the non exposed cohort  a) drawn from the same community as the exposed cohort  b) drawn from a different source  c) no description of the derivation of the non exposed cohort | Ascertainment of exposure  a) secure record (eg surgical records)  b) structured interview  c) written self report  d) no description | Demonstration that outcome of interest was not present at start of study  a) yes  b) no | Comparability of cohorts on the basis of the design or analysis  a) study controls for _____________ (select the most important factor)  b) study controls for any additional factor | Assessment of outcome  a) independent blind assessment  b) record linkage  c) self report  d) no description | Was follow-up long enough for outcomes to occur  a) yes (select an adequate follow up period for outcome of interest)  b) no | Adequacy of follow up of cohorts  a) complete follow up - all subjects accounted for    b) subjects lost to follow up unlikely to introduce bias - small number lost - > ____ % (select an  adequate %) follow up, or description provided of those lost)  c) follow up rate < ____% (select an adequate %) and no description of those lost  d) no statement | Score |
| --- | --- | --- | --- | --- | --- | --- | --- | --- | --- |
| Cayuelas,  2019[7] | 1 | 1 | 1 | 1 | 2 | 1 | 0 | 1 | 8 |
| Fernandez, 2016[8] | 1 | 0 | 0 | 1 | 2 | 0 | 0 | 1 | 5 |
| Golden, 2015[9] | 1 | 0 | 1 | 1 | 1 | 1 | 0 | 0 | 5 |
| Goulet, 2014[10] | 1 | 0 | 1 | 1 | 2 | 1 | 1 | 1 | 8 |
| Jichlinski, 2018[11] | 1 | 0 | 1 | 1 | 2 | 1 | 1 | 1 | 8 |
| Joore, 2016[12] | 1 | 0 | 1 | 1 | 2 | 0 | 1 | 1 | 6 |
| Kapadia, 2018[13] | 1 | 0 | 1 | 1 | 2 | 1 | 0 | 1 | 7 |
| Katz,  2016[14] | 1 | 0 | 1 | 1 | 2 | 1 | 0 | 1 | 7 |
| Klein,  2011[15] | 1 | 0 | 1 | 1 | 2 | 1 | 0 | 1 | 7 |
| Lanier, 2014[16] | 1 | 0 | 0 | 1 | 1 | 0 | 0 | 0 | 3 |
| Mullens, 2019[17] | 1 | 0 | 1 | 1 | 2 | 1 | 0 | 1 | 7 |
| Owusu-Edusei Jr, 2015[18] | 1 | 0 | 0 | 1 | 2 | 0 | 0 | 0 | 4 |
| Petlo,  2011[19] | 1 | 0 | 1 | 1 | 2 | 1 | 0 | 1 | 7 |
| Petsis, 2020[20] | 1 | 0 | 1 | 1 | 2 | 1 | 0 | 0 | 6 |
| Ruutel, 2018[21] | 1 | 0 | 1 | 1 | 2 | 1 | 0 | 1 | 7 |
| Schechter, 2017[22] | 1 | 0 | 1 | 1 | 2 | 1 | 1 | 1 | 8 |
| Selvey, 2018[23] | 1 | 0 | 1 | 1 | 2 | 1 | 1 | 1 | 8 |
| Snow, 2011[24] | 1 | 0 | 1 | 1 | 2 | 1 | 1 | 1 | 8 |
| Wang, 2008[25] | 1 | 1 | 1 | 1 | 2 | 1 | 1 | 1 | 9 |
| Ward, 2016[26] | 1 | 0 | 0 | 1 | 2 | 0 | 0 | 1 | 5 |
| Waxman, 2016[27] | 1 | 0 | 1 | 1 | 2 | 1 | 1 | 1 | 8 |
| Wood, 2014[28] | 1 | 0 | 1 | 1 | 1 | 1 | 0 | 0 | 5 |
| Youssef, 2018[29] | 1 | 1 | 1 | 1 | 2 | 1 | 1 | 1 | 9 |
| Yumori, 2021[30] | 1 | 0 | 1 | 1 | 2 | 1 | 0 | 1 | 7 |

**Newcastle-Ottawa Scale – Cross-sectional Studies**

| **Authors** | Representativeness of the sample:  a. Truly representative of the average in the target population. * (all subjects or random sampling)  b. Somewhat representative of the average in the target group. * (non-random sampling)  c. Selected group of users/convenience sample.  d. No description of the derivation of the included subjects. | Sample size  :  a. Justified and satisfactory (including sample size calculation). *  b. Not justified.  c. No information provided | Non-respondents:  a. Proportion of target sample recruited attains pre-specified target or basic summary of non-respondent characteristics in sampling frame recorded. *  b. Unsatisfactory recruitment rate, no summary data on non-respondents.  c. No information provided | Ascertainment of the exposure (risk factor):  a. Vaccine records/vaccine registry/clinic registers/hospital records only. **  b. Parental or personal recall and vaccine/hospital records. *  c. Parental/personal recall only. | Comparability of subjects in different outcome groups on the basis of design or analysis. Confounding factors controlled.  a. Data/ results adjusted for relevant predictors/risk factors/confounders e.g. age, sex, time since vaccination, etc. **  b. Data/results not adjusted for all relevant confounders/risk factors/information not provided. | Assessment of outcome:  a. Independent blind assessment using objective validated laboratory methods. **  b. Unblinded assessment using objective validated laboratory methods. **  c. Used non-standard or non-validated laboratory methods with gold standard. *  d. No description/non-standard laboratory methods used. | Statistical test:  a. Statistical test used to analyse the data clearly described, appropriate and measures of association presented including confidence intervals and probability level (p value). *  b. Statistical test not appropriate, not described or incomplete. | Score |
| --- | --- | --- | --- | --- | --- | --- | --- | --- |
| Adam, 2014[31] | 1 | 1 | 1 | 1 | 2 | 0 | 1 | 7 |
| Adekeye, 2016[32] | 1 | 1 | 0 | 2 | 2 | 0 | 1 | 7 |
| Assi, 2019[33] | 1 | 1 | 1 | 1 | 2 | 2 | 1 | 9 |
| Avoundjian, 2019[34] | 1 | 1 | 0 | 2 | 0 | 2 | 1 | 7 |
| Badman, 2016[35] | 1 | 1 | 0 | 2 | 2 | 2 | 1 | 9 |
| Baker, 2015[36] | 1 | 1 | 1 | 1 | 0 | 0 | 1 | 5 |
| Balira, 2015[37] | 1 | 1 | 1 | 1 | 0 | 1 | 0 | 5 |
| Banerjee, 2020[38] | 1 | 1 | 1 | 2 | 0 | 1 | 1 | 7 |
| Barber, 2011[39] | 1 | 1 | 1 | 1 | 0 | 0 | 1 | 5 |
| Barnes, 2019[40] | 1 | 1 | 1 | 2 | 2 | 0 | 1 | 8 |
| Bauermeister, 2015[41] | 0 | 1 | 0 | 1 | 0 | 0 | 1 | 3 |
| Bradley, 2013[42] | 1 | 1 | 1 | 1 | 2 | 0 | 1 | 7 |
| Bremer, 2016[43] | 1 | 1 | 1 | 1 | 0 | 0 | 1 | 5 |
| Bristow, 2018[44] | 0 | 1 | 0 | 0 | 1 | 1 | 1 | 4 |
| Carcamo, 2012[45] | 1 | 1 | 1 | 2 | 2 | 2 | 1 | 10 |
| Chen J, 2011[46] | 1 | 1 | 0 | 2 | 2 | 0 | 1 | 7 |
| Chow E, 2018[47] | 1 | 1 | 1 | 2 | 2 | 2 | 1 | 10 |
| Cushman, 2019[48] | 1 | 1 | 1 | 1 | 0 | 0 | 1 | 5 |
| Gamagedara, 2014[49] | 1 | 1 | 1 | 2 | 2 | 2 | 1 | 10 |
| Gilbert, 2018[50] | 1 | 1 | 1 | 2 | 2 | 0 | 1 | 8 |
| Goyal, 2013[51] | 1 | 1 | 1 | 2 | 2 | 2 | 1 | 10 |
| Heard, 2020[52] | 1 | 1 | 1 | 1 | 0 | 0 | 1 | 5 |
| Inghels, 2020[53] | 1 | 1 | 1 | 1 | 2 | 0 | 1 | 7 |
| Josten, 2018[54] | 1 | 0 | 0 | 1 | 0 | 0 | 0 | 2 |
| Kharsany, 2010[55] | 1 | 1 | 1 | 1 | 0 | 0 | 0 | 4 |
| Kilmarx, 2018[56] | 1 | 1 | 0 | 1 | 2 | 2 | 1 | 8 |
| Klein, 2014[57] | 1 | 1 | 1 | 2 | 2 | 2 | 1 | 10 |
| Li, J, 2016[58] | 1 | 1 | 0 | 1 | 0 | 2 | 1 | 6 |
| Llata, 2018[59] | 1 | 1 | 1 | 2 | 2 | 2 | 1 | 10 |
| Lopez, 2019[60] | 1 | 1 | 0 | 2 | 0 | 2 | 1 | 7 |
| MacDonald, 2010[61] | 1 | 1 | 1 | 2 | 0 | 0 | 0 | 5 |
| Marsh, 2013[62] | 1 | 1 | 0 | 2 | 0 | 0 | 0 | 4 |
| Maxwell, 2017[63] | 1 | 1 | 0 | 1 | 1 | 0 | 1 | 5 |
| Mohammed, 2017[64] | 1 | 1 | 1 | 2 | 2 | 0 | 1 | 8 |
| Moore, 2016[65] | 1 | 1 | 1 | 1 | 2 | 0 | 1 | 7 |
| Moore, 2013[66] | 1 | 1 | 1 | 1 | 2 | 0 | 1 | 7 |
| Muhindo, 2019[67] | 1 | 1 | 0 | 1 | 2 | 0 | 1 | 6 |
| Muhindo, 2020[68] | 1 | 1 | 1 | 1 | 2 | 0 | 1 | 7 |
| Murtaugh, 2020[69] | 1 | 1 | 1 | 2 | 2 | 0 | 1 | 8 |
| Ngo, 2013[70] | 1 | 1 | 0 | 1 | 2 | 1 | 1 | 6 |
| Pai, 2012[71] | 1 | 1 | 1 | 2 | 2 | 2 | 1 | 10 |
| Rocchetti, 2015[72] | 1 | 1 | 1 | 1 | 0 | 0 | 1 | 5 |
| Saunders, 2012[73] | 1 | 1 | 0 | 1 | 0 | 0 | 1 | 4 |
| Sullivan, 2021[74] | 0 | 1 | 1 | 2 | 1 | 0 | 1 | 6 |
| Tobin-West, 2013[75] | 1 | 1 | 1 | 1 | 1 | 0 | 1 | 6 |
| Tucker, 2012[76] | 0 | 1 | 1 | 1 | 2 | 0 | 1 | 6 |
| Tucker, 2012[77] | 0 | 1 | 1 | 1 | 2 | 2 | 1 | 8 |
| Tucker, 2011[78] | 0 | 1 | 1 | 1 | 2 | 1 | 1 | 7 |
| Wang Cheng, 2019[79] | 0 | 1 | 1 | 0 | 2 | 2 | 1 | 7 |
| Williford, 2021[80] | 1 | 1 | 1 | 2 | 2 | 2 | 1 | 10 |
| Zhao, 2020[81] | 0 | 1 | 1 | 2 | 2 | 2 | 1 | 9 |

**Newcastle-Ottawa Scale – Case-control Studies**

| **Authors** | Is the case definition adequate?  a) yes, with independent validation  b) yes, eg record linkage or based on self reports  c) no description | Representativeness of the cases  a) consecutive or obviously representative series of cases  b) potential for selection biases or not stated | Selection of Controls  a) community controls  b) hospital controls  c) no description | Definition of Controls  a) no history of disease (endpoint)  b) no description of source | Comparability of cases and controls on the basis of the design or analysis  a) study controls for _______________ (Select the most important factor.)    b) study controls for any additional factor Ø (This criteria could be modified to indicate specific  control for a second important factor.) | Assessment of exposure  a) secure record (eg surgical records)  b) structured interview where blind to case/control status  c) interview not blinded to case/control status  d) written self report or medical record only  e) no description | Same method of ascertainment for cases and controls  a) yes  b) no. | Non-Response rate  a) same rate for both groups  b) non respondents described  c) rate different and no designation | Score |
| --- | --- | --- | --- | --- | --- | --- | --- | --- | --- |
| Joore, 2016[12] | 1 | 1 | 1 | 1 | 2 | 1 | 1 | 0 | 8 |

| Author | 1. Is there congruity between the stated philosophical perspective and the research methodology? | 2. Is there congruity between the research methodology and the research question or objectives? | 3. Is there congruity between the research methodology and the methods used to collect data? | 4. Is there congruity between the research methodology and the representation and analysis of data? | 5. Is there congruity between the research methodology and the interpretation of results? | 6. Is there a statement locating the researcher culturally or theoretically? | 7. Is the influence of the researcher on the research, and vice- versa, addressed? | 8. Are participants, and their voices, adequately represented? | 9. Is the research ethical according to current criteria or, for recent studies, and is there evidence of ethical approval by an appropriate body? | 10. Do the conclusions drawn in the research report flow from the analysis, or interpretation, of the data? |
| --- | --- | --- | --- | --- | --- | --- | --- | --- | --- | --- |
| **Baker, 2015[82]** | Unclear | Y | Y | Y | Y | N | N | Y | Y | Y |
| **Balan, 2020[83]** | Unclear | Y | Y | Y | Y | N | N | Y | Y | Y |
| **Bien, 2015[84]** | Unclear | Y | Y | Y | Y | N | N | Y | Unclear | Y |
| **Bradley, 2013[42]** | Unclear | Y | Y | Y | Y | N | N | Y | N | Y |
| **Jones, 2017[85]** | Y | Y | Y | Y | Y | N | N | Y | Y | Y |
| **Hottes, 2012[86]** | Unclear | Y | Y | Y | Y | N | N | Y | Y | Y |
| **Joore, 2017[87]** | Unclear | Y | Y | Y | Y | N | N | Y | N | Y |
| **Knight, 2012[88]** | Y | Y | Y | Y | Y | Y | N | Y | Y | Y |
| **Lanier, 2014[16]** | Y | Y | Y | Y | Y | N | N | Y | N | Y |
| **McDonagh, 2019[89]** | Unclear | Y | Y | Y | Y | N | N | Unclear | N | Y |
| **Mullens, 2019[17]** | Unclear | Y | Y | Y | Y | N | N | Y | Y | Y |
| **Phrasisombath, 2012[90]** | Unclear | Y | Y | Y | Y | N | N | Y | Y | Y |
| **Scheim, 2016[91]** | Unclear | Y | Y | Y | Y | N | N | Y | Y | Y |
| **Slinkard, 2011[92]** | Unclear | Y | Y | Y | Y | N | N | Y | Y | Y |
| **Sullivan, 2021[93]** | Unclear | Y | Y | Y | Y | N | N | Y | Y | Y |
| **Underhill, 2014[94]** | Unclear | Y | Y | Y | Y | N | N | Y | Y | Y |

**REFERENCES**

1. Beck E, Armbruster B, Birkett M, Mustanski B. The value of timely implementation in HIV/STI testing: Cost-effectiveness vs. Speed of intervention rollout. Value in Health. 2016;19 (7):A365.

2. Owusu-Edusei Jr K, Tao G, Gift TL, Wang A, Wang L, Tun Y, et al. Cost-effectiveness of Integrated Routine Offering of Prenatal HIV and Syphilis Screening in China. Sexually Transmitted Diseases. 2014;41(2):103-10.

3. Prabhu VS, Farnham PG, Hutchinson AB, Soorapanth S, Heffelfinger JD, Golden MR, et al. Cost-effectiveness of HIV screening in STD clinics, emergency departments, and inpatient units: a model-based analysis. PLoS ONE [Electronic Resource].6(5):e19936.

4. Bauermeister J, Pingel E, Jadwin-Cakmak L, Harper G, Horvath K, Weiss G, et al. Acceptability and Preliminary Efficacy of a Tailored Online HIV/STI Testing Intervention for Young Men who have Sex with Men: The Get Connected! Program. AIDS & Behavior. 2015;19(10):1860-74.

5. Brown L, Patel S, Ives NJ, McDermott C, Ross JD. Is non-invasive testing for sexually transmitted infections an efficient and acceptable alternative for patients? A randomised controlled trial. Sexually Transmitted Infections.86(7):525-31.

6. Sharma A, Kahle E, Todd K, Peitzmeier S, Stephenson R. Variations in Testing for HIV and Other Sexually Transmitted Infections Across Gender Identity Among Transgender Youth. Transgender Health.4(1):46-57.

7. Cayuelas Redondo L, Ruiz M, Kostov B, Sequeira E, Noguera P, Herrero MA, et al. Indicator condition-guided HIV testing with an electronic prompt in primary healthcare: a before and after evaluation of an intervention. Sexually Transmitted Infections. 2019;95(4):238-43.

8. Fernandez-Balbuena S, Hoyos J, Rosales-Statkus ME, Nardone A, Vallejo F, Ruiz M, et al. Low HIV testing uptake following diagnosis of a sexually transmitted infection in Spain: implications for the implementation of efficient strategies to reduce the undiagnosed HIV epidemic. AIDS Care. 2016;28(6):677-83.

9. Golden MR, Katz DA, Kern D, Heal D, Kerani R, Dombrowski JC. Sexually transmitted disease partner services increase HIV testing among men who have sex with men. Topics in Antiviral Medicine. 2015;23 (E-1):507.

10. Goulet JL, Martinello RA, Bathulapalli H, Higgins D, Driscoll MA, Brandt CA, et al. STI diagnosis and HIV testing among OEF/OIF/OND veterans. Medical Care.52(12):1064-7.

11. Jichlinski A, Goyal MK, Badolato GM, Pastor W. Rates of HIV and Syphilis Testing Among Adolescents Diagnosed with Pelvic Inflammatory Disease. Pediatrics. 2018;142.

12. Joore IK, Twisk DE, Vanrolleghem AM, de Ridder M, Geerlings SE, van Bergen JEAM, et al. The need to scale up HIV indicator condition-guided testing for early case-finding: a case-control study in primary care. BMC Fam Pract. 2016;17(1):161-.

13. Kapadia SN, Singh HK, Jones S, Merrick S, Vaamonde CM. Missed Opportunities for HIV Testing of Patients Tested for Sexually Transmitted Infections at a Large Urban Health Care System From 2010 to 2015. Open Forum Infectious Diseases.5(7):ofy165.

14. Katz DA, Dombrowski JC, Kerani RP, Aubin MR, Kern DA, Heal DD, et al. Integrating HIV Testing as an Outcome of STD Partner Services for Men Who Have Sex with Men. AIDS Patient Care & Stds.30(5):208-14.

15. Klein P, Bishop A, Leone P. Hiv testing of patients receiving an std evaluation in a north carolina community health center. Sexually Transmitted Infections. 2011;87:A208.

16. Lanier Y, Castellanos T, Barrow RY, Jordan WC, Caine V, Sutton MY. Brief sexual histories and routine HIV/STD testing by medical providers. AIDS Patient Care & Stds.28(3):113-20.

17. Mullens AB, Duyker J, Brownlow C, Lemoire J, Daken K, Gow J. Point-of-care testing (POCT) for HIV/STI targeting MSM in regional Australia at community 'beat' locations. BMC Health Services Research.19(1):93.

18. Owusu-Edusei Jr K, Gift TL, Patton ME, Johnson DB, Valentine JA, Owusu-Edusei K, Jr. Estimating the Total Annual Direct Cost of Providing Sexually Transmitted Infection and HIV Testing and Counseling for Men Who Have Sex With Men in the United States. Sexually Transmitted Diseases. 2015;42(10):586-9.

19. Petlo T, Fairley CK, Whitton B, Coles K, Chen MY. HIV-testing of men who have sex with men: variable testing rates among clinicians. (Special Issue: HIV testing.). International Journal of STD & AIDS. 2011;22(12):727-9.

20. Petsis D, Jungwon M, Yuan-Shung VH, Akers AY, Wood S. HIV Testing Among Adolescents With Acute Sexually Transmitted Infections. Pediatrics. 2020;145(4):1-8.

21. Rüütel K, Lemsalu L, Lätt S, Opt TbH. Monitoring HIV‐indicator condition guided HIV testing in Estonia. HIV Medicine. 2018;19:47-51.

22. Schechter SB, Romo DL, Cohall AT, Neu NM. Approach to Human Immunodeficiency Virus/Sexually Transmitted Infection Testing for Men at an Urban Urgent Care Center. Sexually Transmitted Diseases.44(4):255-9.

23. Selvey LA, Slimings C, Adams E, Manuel J. Incidence and predictors of HIV, chlamydia and gonorrhoea among men who have sex with men attending a peer-based clinic. Sexual Health. 2018;15(5):451-9.

24. Snow AF, Cummings R, Owen L, El-Hyak C, Hellard ME, Vodstrcil L, et al. Introduction of a sexual health practice nurse increases sti testing among MSM in general practice. Sexually Transmitted Infections. 2011;87:A98.

25. Wang B, Li X, Stanton B, McGuire J. Correlates of HIV/STD testing and willingness to test among rural-to-urban migrants in China. AIDS & Behavior.14(4):891-903.

26. Ward JS, Dyda A, McGregor S, Rumbold A, Garton L, Donovan B, et al. Low HIV testing rates among people with a sexually transmissible infection diagnosis in remote Aboriginal communities. Medical Journal of Australia.205(4):168-71.

27. Waxman M, Ata A, Frisch A, Sutton L. Rates of emergency department human immunodeficiency virus (HIV) tested in patients tested for sexually transmitted diseases. Open Forum Infectious Diseases Conference: ID Week. 2016;3(Supplement 1).

28. Wood M, Ellks R, Grobicki M. Outreach sexual infection screening and postal tests in men who have sex with men: are they comparable to clinic screening? International Journal of STD & AIDS.26(6):428-31.

29. Youssef E, Sanghera T, Bexley A, Hayes M, Perry N, Dosekun O, et al. HIV testing in patients presenting with indicator conditions in outpatient settings: offer and uptake rates, and educational and active interventions. International Journal of STD & AIDS. 2018;29(13):1289-94.

30. Yumori C, Zucker J, Theodore D, Chang M, Carnevale C, Slowikowski J, et al. Women Are Less Likely to Be Tested for HIV or Offered Preexposure Prophylaxis at the Time of Sexually Transmitted Infection Diagnosis. Sexually Transmitted Diseases.48(1):32-6.

31. Adam P, Wit J, Bourne C, Knox D, Purchas J. Promoting Regular Testing: An Examination of HIV and STI Testing Routines and Associated Socio-Demographic, Behavioral and Social-Cognitive Factors Among Men Who have Sex with Men in New South Wales, Australia. AIDS & Behavior. 2014;18(5):921-32.

32. Adekeye OA, Abara WE, Xu J, Lee JM, Rust G, Satcher D. HIV Screening Rates among Medicaid Enrollees Diagnosed with Other Sexually Transmitted Infections. PLoS One. 2016;11(8):e0161560-e.

33. Assi A, Abu Zaki S, Ghosn J, Kinge N, Naous J, Ghanem A, et al. Prevalence of HIV and other sexually transmitted infections and their association with sexual practices and substance use among 2238 MSM in Lebanon. Scientific Reports.9(1):15142.

34. Avoundjian T, Stewart J, Peyton D, Lewis C, Johnson K, Glick SN, et al. Integrating HIV testing into syphilis partner services in Mississippi to improve HIV case finding. Sexually Transmitted Diseases. 2018;03.

35. Badman SG, Vallely LM, Toliman P, Kariwiga G, Lote B, Pomat W, et al. A novel point-of-care testing strategy for sexually transmitted infections among pregnant women in high-burden settings: results of a feasibility study in Papua New Guinea. BMC Infectious Diseases.16:250.

36. Baker U, Okuga M, Waiswa P, Manzi F, Peterson S, Hanson C, et al. Bottlenecks in the implementation of essential screening tests in antenatal care: Syphilis, HIV, and anemia testing in rural Tanzania and Uganda. International Journal of Gynaecology & Obstetrics.130 Suppl 1:S43-50.

37. Balira R, Mabey D, Weiss H, Ross DA, Changalucha J, Watson-Jones D. The need for further integration of services to prevent mother-to-child transmission of HIV and syphilis in Mwanza City, Tanzania. (Special Issue: Accelerating dual elimination of mother-to-child transmission of syphilis and HIV through use of new diagnostic tools.). International Journal of Gynecology & Obstetrics. 2015;130(Suppl. 1):S51-S7.

38. Banerjee P, Madhwapathi V, Thorley N, Radcliffe K. A service evaluation comparing home-based testing to clinic-based testing for HIV, syphilis and hepatitis B in Birmingham and Solihull. International Journal of STD & AIDS.31(7):613-8.

39. Barber B, Hellard M, Jenkinson R, Spelman T, Stoove M. Sexual history taking and sexually transmissible infection screening practices among men who have sex with men: a survey of Victorian general practitioners. Sexual Health.8(3):349-54.

40. Barnes A, Jetelina KK, Betts AC, Mendoza T, Pranavi S, Tiro JA. Emergency department testing patterns for sexually transmitted diseases in North Texas. Sexually Transmitted Diseases. 2019;46(7):434-9.

41. Bauermeister J, Pingel E, Jadwin-Cakmak L, Meanley S, Alapati D, Moore M, et al. The Use of Mystery Shopping for Quality Assurance Evaluations of HIV/STI Testing Sites Offering Services to Young Gay and Bisexual Men. AIDS & Behavior. 2015;19(10):1919-27.

42. Bradley H, Asbel L, Bernstein K, Mattson M, Pathela P, Mohamed M, et al. HIV Testing Among Patients Infected with Neisseria gonorrhoeae: STD Surveillance Network, United States, 2009-2010. AIDS & Behavior. 2013;17(3):1205-10.

43. Bremer V, Haar K, Gassowski M, Hamouda O, Nielsen S. STI tests and proportion of positive tests in female sex workers attending local public health departments in Germany in 2010/11. BMC Public Health.16(1):1175.

44. Bristow CC, Kojima N, Lee S, Leon SR, Ramos LB, Konda KA, et al. HIV and syphilis testing preferences among men who have sex with men and among transgender women in Lima, Peru. Plos One. 2018;13(10).

45. Cárcamo CP, Campos PE, García PJ, Hughes JP, Garnett GP, Holmes KK, et al. Prevalences of sexually transmitted infections in young adults and female sex workers in Peru: a national population-based survey. Lancet Infectious Diseases. 2012;12(10):765-73.

46. Chen JY, Ma Q, Everhard F, Yermilov I, Tian H, Mayer KH. HIV screening in commercially insured patients screened or diagnosed with sexually transmitted diseases or blood-borne pathogens. Sexually Transmitted Diseases.38(6):522-7.

47. Chow EPF, Fortune R, Dobinson S, Wakefield T, Read TRH, Chen MY, et al. Evaluation of the Implementation of a New Nurse-Led Express "Test-And-Go" Human Immunodeficiency Virus/Sexually Transmitted Infection Testing Service for Men Who Have Sex With Men at a Sexual Health Center in Melbourne, Australia. Sexually Transmitted Diseases.45(6):429-34.

48. Cushman TA, Graves SK, Little SJ. Attitudes and Preferences Regarding the Use of Rapid Self-Testing for Sexually Transmitted Infections and HIV in San Diego Area Men Who Have Sex With Men. Open Forum Infectious Diseases.6(3):ofz043.

49. Gamagedara N, Dobinson S, Cummings R, Fairley CK, Lee D. An evaluation of an express testing service for sexually transmissible infections in low-risk clients without complications. Sexual Health.11(1):37-41.

50. Gilbert M, Thomson K, Salway T, Haag D, Grennan T, Fairley CK, et al. Differences in experiences of barriers to STI testing between clients of the internet-based diagnostic testing service GetCheckedOnline.com and an STI clinic in Vancouver, Canada. Sexually Transmitted Infections.95(2):151-6.

51. Goyal M, Witt R, Gerber J, Hayes K, Zaoutis T. Physician adherence to sexual health and STI/HIV screening recommendations during routine adolescent health visits. Journal of Adolescent Health. 2013;52(2):S84.

52. Heard E, Oost E, McDaid L, Mutch A, Dean J, Fitzgerald L. How can HIV/STI testing services be more accessible and acceptable for gender and sexually diverse young people? A brief report exploring young people's perspectives in Queensland. Health Promotion Journal of Australia.31(1):150-5.

53. Inghels M, Kouassi AK, Niangoran S, Bekelynck A, Carillon S, Sika L, et al. Cascade of Provider-Initiated Human Immunodeficiency Virus Testing and Counselling at Specific Life Events (Pregnancy, Sexually Transmitted Infections, Marriage) in Cote d'Ivoire. Sexually Transmitted Diseases. 2020;47(1):54-61.

54. Josten MS, Keeshin S. Knowledge, practices, and attitudes of youth providers about STI, HIV testing, and prep. Open Forum Infectious Diseases. 2018;5 (Supplement 1):S697.

55. Kharsany AB, Karim QA, Karim SS. Uptake of provider-initiated HIV testing and counseling among women attending an urban sexually transmitted disease clinic in South Africa - missed opportunities for early diagnosis of HIV infection. AIDS Care.22(5):533-7.

56. Kilmarx PH, Gonese E, Lewis DA, Chirenje ZM, Barr BAT, Latif AS, et al. HIV infection in patients with sexually transmitted infections in Zimbabwe - Results from the Zimbabwe STI etiology study. PLoS ONE [Electronic Resource].13(6):e0198683.

57. Klein PW, Martin IBK, Quinlivan EB, Gay CL, Leone PA. Missed opportunities for concurrent HIV-STD testing in an academic emergency department. (Special Issue: Program collaboration and service integration in the prevention and control of HIV infection, viral hepatitis, STDs, and tuberculosis in the U.S.: lessons learned from the field.). Public Health Reports. 2014;129(1 (Suppl.1):12-20.

58. Li J, Jiang N, Yue X, Gong X. [HIV detection and prevalence among sexullay transmitted diseases clinic patients in seven provinces (Autonomous Region)]. Chung-Hua Liu Hsing Ping Hsueh Tsa Chih Chinese Journal of Epidemiology.37(3):358-61.

59. Llata E, Braxton J, Asbel L, Kerani RP, Murphy R, Pugsley R, et al. New Human Immunodeficiency Virus Diagnoses Among Men Who Have Sex With Men Attending Sexually Transmitted Disease Clinics, STD Surveillance Network, January 2010 to June 2013. Sexually Transmitted Diseases.45(9):577-82.

60. Lopez R, Hustey FM, Schold JD, Seballos SS, Phelan MP. Emergency Department Sexually Transmitted Infection Testing and Compliance With CDC HIV Testing Guidelines in a National Sample of Emergency Departments. Annals of Emergency Medicine. 2019;74(4):S95-S.

61. MacDonald R, Goodall L, Nair V, Baguley S, Clutterbuck D. Completion of a British Association for Sexual Health and HIV regional audit loop: HIV testing in genitourinary medicine clinics in Scotland in 2004 and 2008. International Journal of STD & AIDS.21(9):648-9.

62. Marsh K, Chan S, Wheatley N, Duffell S, Lau R, Hughes G. Missed STI and HIV testing opportunities among male prisoners in England. Sexually Transmitted Infections Conference: STI and AIDS World Congress. 2013;89(SUPPL. 1).

63. Maxwell S. General Practitioners' views and experiences on the barriers and facilitators that men who have sex with men have when accessing primary care for HIV testing and sexual health screening. Primary Health Care Research & Development.19(2):205-9.

64. Mohammed H, Dabrera G, Furegato M, Yin Z, Nardone A, Hughes G. Refusal of HIV testing among black Africans attending sexual health clinics in England, 2014: a review of surveillance data. Sexually Transmitted Infections.93(3):217-20.

65. Moore MJ, Barr E, Wilson K, Griner S. Support for Offering Sexual Health Services Through School-Based Health Clinics. Journal of School Health.86(9):660-8.

66. Moore EW. Human immunodeficiency virus and chlamydia/gonorrhea testing among heterosexual college students: who is getting tested and why do some not? Journal of American College Health.61(4):196-202.

67. Muhindo R, Castelnuovo B, Mujugira A, Parkes-Ratanshi R, Sewankambo NK, Kiguli J, et al. Psychosocial correlates of regular syphilis and HIV screening practices among female sex workers in Uganda: a cross-sectional survey. AIDS Research & Therapy [Electronic Resource].16(1):28.

68. Muhindo R, Mujugira A, Castelnuovo B, Sewankambo NK, Parkes-Ratanshi R, Kiguli J, et al. HIV and syphilis testing behaviors among heterosexual male and female sex workers in Uganda. AIDS Research & Therapy [Electronic Resource].17(1):48.

69. Murtaugh KL, Leibowitz A, Chen X, Pourat N. Missed opportunities for HIV screening of new enrollees in California's low income health program. Aids Education and Prevention. 2020;32(1):25-35.

70. Ngo AD, Ha TH, Rule J, Dang CV. Peer-based education and the integration of HIV and sexual and reproductive health services for young people in Vietnam: evidence from a project evaluation. Plos One. 2013;8(11).

71. Pai NP, Kurji J, Singam A, Barick R, Jafari Y, Klein MB, et al. Simultaneous triple point-of-care testing for HIV, syphilis and hepatitis B virus to prevent mother-to-child transmission in India. International Journal of STD & AIDS.23(5):319-24.

72. Rocchetti V, Viard JP. Family practitioners screening for HIV infection. Medecine et Maladies Infectieuses.45(5):157-64.

73. Saunders JM, Mercer CH, Sutcliffe LJ, Hart GJ, Cassell J, Estcourt CS. Where do young men want to access STI screening? A stratified random probability sample survey of young men in Great Britain. Sexually Transmitted Infections.88(6):427-32.

74. Sullivan PS, Lyons MS, Czarnogorski M, Branson BM. Special Issue: Routine screening for HIV infection in medical care settings: a decade of progress and next opportunities. (Special Issue: Routine screening for HIV infection in medical care settings: a decade of progress and next opportunities.). Public Health Reports. 2016;131(Suppl.1):1-146.

75. Tobin-West CI, Lawson AM. Stigma and underutilization of facility-based sexually transmitted infection services undermine human immunodeficiency virus testing in rural communities of Rivers State, Nigeria. International Journal of Health and Allied Sciences. 2013;2(2):108-14.

76. Tucker JD, Walensky RP, Yang L-G, Yang B, Bangsberg DR, Chen X-S, et al. Expanding provider-initiated HIV testing at STI clinics in China. AIDS Care. 2012;24(10):1316-9.

77. Tucker JD, Yang L, Yang B, Young D, Henderson GE, Huang S, et al. Prior HIV testing among STD patients in Guangdong Province, China: opportunities for expanding detection of sexually transmitted HIV infection. Sexually Transmitted Diseases. 2012;39(3):182-7.

78. Tucker JD, Yang L, Yang B, Zheng H, Chang H, Wang C, et al. A twin response to twin epidemics: integrated HIV/syphilis testing at STI clinics in South China. JAIDS, Journal of Acquired Immune Deficiency Syndromes. 2011;57(5):e106-e11.

79. Wang C, Cheng W, Li C, Tang W, Ong JJ, Smith MK, et al. Syphilis self-testing: a nationwide pragmatic study among men who have sex with men in China. Clinical Infectious Diseases. 2020;70(10):2178-86.

80. Williford SL, Humes E, Greenbaum A, Schumacher CM. HIV Screening Among Gonorrhea-Diagnosed Individuals; Baltimore, Maryland; April 2015 to April 2019. Sexually Transmitted Diseases.48(1):42-8.

81. Zhao P, Tang W, Cheng H, Huang S, Zheng H, Yang B, et al. Uptake of provider-initiated HIV and syphilis testing among heterosexual STD clinic patients in Guangdong, China: results from a cross-sectional study. BMJ Open.10(12):e041503.

82. Baker A, Fleury C, Clarke E, Foley E, Samraj S, Rowen D, et al. Increasing screening frequency in men who have sex with men: impact of guidance on risk profiling on workload and earlier diagnosis of sexually transmitted infection and HIV. International Journal of STD & AIDS.24(8):613-7.

83. Balan IC, Lopez-Rios J, Nayak S, Lentz C, Arumugam S, Kutner B, et al. SMARTtest: A Smartphone App to Facilitate HIV and Syphilis Self- and Partner-Testing, Interpretation of Results, and Linkage to Care. AIDS & Behavior.24(5):1560-73.

84. Bien CH, Muessig KE, Lee R, Lo EJ, Yang L, Yang B, et al. HIV and syphilis testing preferences among men who have sex with men in South China: a qualitative analysis to inform sexual health services. Plos One. 2015;10(4).

85. Jones LF, Ricketts E, Town K, Rugman C, Lecky D, Folkard K, et al. Chlamydia and HIV testing, contraception advice, and free condoms offered in general practice: a qualitative interview study of young adults' perceptions of this initiative. British Journal of General Practice.67(660):e490-e500.

86. Hottes TS, Farrell J, Bondyra M, Haag D, Shoveller J, Gilbert M. Internet-based HIV and sexually transmitted infection testing in British Columbia, Canada: opinions and expectations of prospective clients. Journal of Medical Internet Research.14(2):e41.

87. Joore IK, Roosmalen SLv, Bergen JEAMv, Dijk Nv. General practitioners' barriers and facilitators towards new provider-initiated HIV testing strategies: a qualitative study. International Journal of STD & AIDS. 2017;28(5):459-66.

88. Knight R, Shoveller JA, Oliffe JL, Gilbert M, Goldenberg S. Heteronormativity hurts everyone: experiences of young men and clinicians with sexually transmitted infection/HIV testing in British Columbia, Canada. Health: an Interdisciplinary Journal for the Social Study of Health, Illness & Medicine.17(5):441-59.

89. McDonagh L, Omran L, Curtis T, Pach S, Saunders J, Cassell J, et al. 'you feel invisible': A qualitative exploration of young LGBT+ people's attitudes towards STI/HIV testing in primary care. Sexually Transmitted Infections. 2019;95 (Supplement 1):A63.

90. Phrasisombath K, Thomsen S, Sychareun V, Faxelid E. Care seeking behaviour and barriers to accessing services for sexually transmitted infections among female sex workers in Laos: a cross-sectional study. BMC Health Services Research. 2012;12.

91. Scheim AI, Travers R. Barriers and facilitators to HIV and sexually transmitted infections testing for gay, bisexual, and other transgender men who have sex with men. AIDS Care.29(8):990-5.

92. Slinkard MS, Kazer MW. Older adults and HIV and STI screening: the patient perspective. Geriatric Nursing.32(5):341-9.

93. Sullivan SP, Sullivan PS, Stephenson R. Acceptability and Feasibility of a Telehealth Intervention for STI Testing Among Male Couples. Aids and Behavior.

94. Underhill K, Morrow KM, Colleran CM, Holcomb R, Operario D, Calabrese SK, et al. Access to healthcare, HIV/STI testing, and preferred pre-exposure prophylaxis providers among men who have sex with men and men who engage in street-based sex work in the US. PLoS ONE [Electronic Resource].9(11):e112425.
